# Exploring clusters based on ultrasound-detected inflammation in patients with psoriatic arthritis: a post-hoc analysis from the ULTIMATE trial

**DOI:** 10.1186/s12891-025-09434-w

**Published:** 2026-01-30

**Authors:** Maria Antonietta D’Agostino, Philip G Conaghan, Corine Gaillez, Maarten Boers, Esperanza Naredo, Peter Mandl, Philippe Carron, Alejandra López-Rdz, Ruben Burgos-Vargas, Javier Rosa, Catherine Bakewell, Tomas Cazenave, Weibin Bao, David Demanse, Georg Schett

**Affiliations:** 1 heDepartment of Rumatology, Catholic University of Sacred Heart, Roma, Italy; 2 NIHR Leeds Biomedical Research Centre, University of Leeds, Leeds, UK; 3 Novartis Pharma AG, Basel, Switzerland; 4 Department of Epidemiology and Data Science, Amsterdam Rheumatology and Immunology Center, Amsterdam UMC, Vrije Universiteit, Amsterdam, The Netherlands; 5 Department of Rheumatology and Joint and Bone Research Unit, Hospital Fundación, Jiménez Díaz and Autónoma University, Madrid, Spain; 6 Division of Rheumatology, Medical University of Vienna, Vienna, Austria; 7 Department of Rheumatology, University Hospital Ghent, Ghent, Belgium; 8 PSOAPS Psoriasis Clinical and Research Center, Guadalajara, Mexico; 9 Department of Rheumatology, General Hospital of Mexico, Ciudad de Mexico, Mexico; 10 Rheumatology Unit, Internal Medical Services, University Institute, Hospital Italiano de Buenos Aires, Buenos Aires, Argentina; 11 Intermountain Healthcare, Salt Lake City, US; 12 Rheumatology Unit, Instituto de Rehabilitación Psicofísica, Echeverria 955, Buenos Aires, Argentina; 13 Novartis Pharmaceuticals Corporation, East Hanover, US; 14 Department of Internal Medicine 3 – Rheumatology and Immunology, Universitätsklinikum Erlangen, Erlangen, Germany

**Keywords:** Psoriatic arthritis, Synovitis, Ultrasound, Cluster analysis, ULTIMATE trial, Secukinumab

## Abstract

**Background:**

Psoriatic arthritis (PsA) is characterized by heterogeneous musculoskeletal manifestations. The aim of this post-hoc cluster analysis from the ULTIMATE study was to explore whether objective assessment of inflammation, using power-Doppler ultrasound (PDUS), identified homogeneous groups of patients based on the level of severity of ultrasound-detected synovitis and then to determine the longitudinal trajectory of response with secukinumab vs. placebo for each cluster up to week 12.

**Methods:**

The ULTIMATE study enrolled patients with PsA and active clinical and PDUS synovitis. Patients were randomized to either secukinumab 150 or 300 mg, according to psoriasis severity, or placebo for the first 12 weeks. Multiple factor analysis was performed on baseline B-mode and power Doppler (PD) signal core components of the composite Global EULAR-OMERACT synovitis score (GLOESS) at joint level.

**Results:**

Three clusters were identified from all patients enrolled in the ULTIMATE study (*N* = 166), with 49 (30%), 54 (33%), and 63 (38%) patients assigned to clusters 1, 2, and 3, respectively. Higher GLOESS PDUS scores (± standard deviation) were observed in clusters 2 (35 ± 18) and 3 (30 ± 11) than cluster 1 (10 ± 6). While the B-mode core components of GLOESS were higher in clusters 2 (34 ± 18) and 3 (29 ± 11) than cluster 1 (10 ± 6), the PD signal core component was higher in cluster 2 (13 ± 10) than clusters 1 (5 ± 3) and 3 (5 ± 4). Overall, the pattern of distribution of PDUS-detected synovitis showed heterogeneity, with a predominance on small joints in cluster 2, more diffuse across small and large joints in cluster 3 and low frequency in small joints and knees in cluster 1. Change in GLOESS from baseline to week 12 showed a numerical trend toward a treatment difference favoring secukinumab vs. placebo in all three clusters.

**Conclusion:**

Three clusters of patients were identified by ultrasound highlighting the heterogeneity of a typical PsA trial population. The addition of ultrasound to complement clinical assessment of swollen joints may help in better selecting active patients for inclusion in PsA clinical trials, thereby limiting the variability of the therapeutic response usually observed.

**Trial registration:**

ClinicalTrials.gov (identifier: NCT02662985; date: 26/01/2016).

**Supplementary Information:**

The online version contains supplementary material available at 10.1186/s12891-025-09434-w.

## Introduction

The recently updated recommendations from the European Alliance of Associations for Rheumatology (EULAR 2023) [1] and from the International Group for Research and Assessment of Psoriasis and Psoriatic Arthritis (GRAPPA 2021) [2] in psoriatic arthritis (PsA) have highlighted that PsA is a chronic, immune, inflammatory, heterogeneous, and multifaceted disease characterized by seven key clinical manifestations: peripheral arthritis, axial disease, enthesitis, dactylitis, skin and nail psoriasis, and extra-articular manifestations, and underlined the importance of a combined clinical and imaging approach for PsA patients to better control the evolution of the disease [3–7]. Synovitis is a key feature of PsA that may ultimately lead to structural damage and compromised physical function [6].

Suppressing joint inflammation, i.e., synovitis, is an important goal for the treatment of PsA. However, to date, not all patients with clinically active synovitis respond to therapy. The effect of treatment is predominantly assessed by changes in clinical signs and symptoms rather than directly assessing improvement of inflammation at tissue level on joints by imaging.

Little is known about the potential heterogeneity of severity of synovitis detected by ultrasound, and the impact that inflammation detected by imaging at joint level may have on treatment response. A deeper understanding of the true, objective severity of synovitis could be beneficial when assessing the responsiveness of biological treatment.

Power Doppler ultrasound (PDUS), a combination of ultrasonography (US) in B-mode and power Doppler (PD), is a powerful diagnostic tool, not associated with irradiation, which provides good accessibility to peripheral joints and high reproducibility at an overall low cost [8]. It allows objective evaluation of visualization of inflammatory and structural changes to articular and periarticular structures such as the enthesis, as well as structural lesions, such as bone proliferation and erosions [9–13]. The combination of PD and B-mode provides greater details of synovial morphology and blood cell movements, and the use of PD increases sensitivity to low-volume and low‐velocity blood flow at the microvascular level [14].

International organizations such as EULAR and Outcome Measures in Rheumatology (OMERACT) have standardized the use of PDUS for detecting synovitis and developed a composite scoring system at joint and patient level – the Global EULAR-OMERACT Synovitis Score (GLOESS). GLOESS has demonstrated high reliability and responsiveness to treatment [9, 15–18]. The ULTIMATE study (NCT02662985) was the first large, randomized, double-blind, placebo-controlled Phase III study in PsA to use ultrasound inclusion criteria confirming active synovitis; it demonstrated that secukinumab rapidly and significantly decreased synovitis using GLOESS [17].

Advances in machine learning (ML) have opened new possibilities across many fields of medicine, including rheumatology, allowing the detection of patterns in vast amounts of data through ML-based clustering methods with therapeutic or prognostic significance [19–23].

The aim of this exploratory, post-hoc analysis focusing on baseline PDUS characteristics in patients with PsA from the ULTIMATE trial was to perform a cluster analysis to determine homogeneous groups of patients based on the level of severity of joint level PDUS-detected synovitis. The cluster analysis was based only on objective estimation of the severity of synovitis by US at baseline, without including clinical parameters or relying on the distribution of synovitis. Such exploratory cluster analyses generate hypotheses that may guide the design of future clinical trials, for example, the need to select a more homogenous patient population in terms of synovitis. The second exploratory objective was to describe the longitudinal trajectory of treatment with secukinumab vs. placebo for each identified cluster during the 12-week randomized double-blind period.

## Methods

### Study design and patients

The ULTIMATE study was registered at ClinicalTrials.gov (identifier: NCT02662985; registration date: 26/01/2016). Details of the ULTIMATE study design have been reported previously [17]. Briefly, ULTIMATE was a 52-week, Phase III study comprising a 12-week, double-blind, placebo-controlled treatment period followed by a 12-week, open-label treatment period, a 6-month open-label extension period and a 12-week safety follow-up period (Supplementary Fig. S1). The study protocol was reviewed and approved by the independent ethics committee or institutional review board for each participating center and was conducted according to the International Council for Harmonization E6 Guideline for Good Clinical Practice that has its origin in the Declaration of Helsinki. Written informed consent was obtained from all enrolled patients.

The key inclusion criteria were adult patients with active PsA (ClASsification criteria for Psoriatic Arthritis [CASPAR] criteria [24]) defined by clinically tender and swollen joints (at least three of each), active PDUS-detected synovitis according to a pre-defined cut-off, and at least one clinical enthesitis defined by the Spondyloarthritis Research Consortium of Canada (SPARCC) index [25] without any requirement of an active enthesitis as observed on PDUS at screening and baseline. Patients had an inadequate response to conventional synthetic disease-modifying anti-rheumatic drugs (DMARDs) and were naïve to biological DMARDs. Detailed inclusion and exclusion criteria previously published [17] are provided in the Supplementary Appendix.

Patients could continue to receive concomitant methotrexate, glucocorticoids, and nonsteroidal anti-inflammatory drugs (NSAIDs) at a stable standard dose from 1 month prior to screening to 24 weeks [17].

Patients were randomly assigned to receive either weekly subcutaneous secukinumab (300 mg or 150 mg according to the severity of skin disease) or placebo for 4 weeks, followed by 4-weekly dosing at weeks 4 and 8. The primary outcome was the mean change in the ultrasound GLOESS from baseline to week 12 in the secukinumab vs. placebo group. For this analysis, data from the two doses of secukinumab (150 mg and 300 mg) were pooled.

Key secondary endpoints included the American College of Rheumatology (ACR) response criteria, ACR20 and ACR50, at weeks 1, 2, 4, 8, and 12, and SPARCC change from baseline to week 12.

### Ultrasound assessments of joints and entheses

PDUS evaluation of synovitis and enthesitis was performed at screening, baseline, and weeks 1, 2, 4, 8, and 12. The following 24 joints were evaluated bilaterally: metacarpophalangeal (MCP) joint 1–5, proximal interphalangeal (PIP) joint 1–5, metatarsophalangeal (MTP) joint 1–5, distal interphalangeal (DIP) joint 2–5, wrists (all compartments), elbow, shoulder (glenohumeral), knee, and ankle (tibiotalar). Joints were scanned at each visit from the dorsal aspect with the joint in neutral position, except for the knee, which was examined in a semi-flexed position (30°). All recesses of each joint were scanned, and the maximal grade of PDUS synovitis detected was selected to represent the final grade for the given joint. All PDUS evaluations were performed at each site by an expert with more than 5 years of experience in musculoskeletal ultrasound who was blinded to clinical evaluation and therapy. To ensure homogeneity of PDUS synovitis and enthesitis scoring, all ultrasound investigators completed an extensive 2-day training session, including ultrasound examination of patients with PsA and different spectra of disease activity. An ultrasound atlas with representative images for each grading of synovitis and/or enthesitis recorded in each of the examined joints was provided to the sonographer to help them in case of doubt when scoring. In addition, ultrasound settings were not changed during the study; standardized joint, enthesis, and probe positions were used, and software was not upgraded. Centers were advised to create a fixed study setting to be used at each evaluation.

The presence of synovitis according to the EULAR-OMERACT definition was scored with a PDUS composite semi-quantitative scale (0–3) at joint level and its core components (synovial hypertrophy [SH] B-mode and PD signal) at each visit (Supplementary Table S1). The GLOESS at patient level was calculated as the sum of each PDUS composite score for the 24 pairs of joints examined, with a score range of 0–144. As previously reported, GLOESS incorporates both B-mode and PD measures of synovitis and allows for the evaluation of changes in the activity and morphology of synovitis. Further details on PDUS measures of synovitis and grading of severity can be found in the primary manuscript [17].

PDUS has proven to be a valid, sensitive and reliable means of evaluating enthesitis in spondyloarthritis (SpA), showing an abnormal vascularization of entheses, which was exclusively detected in SpA [26, 27]. Using a Delphi process, the OMERACT group developed a reliable enthesitis score at enthesitis level which includes a clear definition of each elementary component [28]. Quantification of entheseal involvement by ultrasound is best undertaken using semi-quantitative scoring systems that combine both inflammatory and structural damage signs for use in clinical studies [29]. Therefore, a total of six targeted pairs of entheses were simultaneously examined bilaterally: common extensor tendon at the lateral humeral epicondyle insertion, quadriceps tendon at its insertion at the superior pole of the patella, patellar tendon at its proximal insertion at the inferior pole of the patella and distal insertion at the tibial tuberosity, Achilles tendon at its insertion at the calcaneus, and plantar aponeurosis at its insertion at the calcaneus. Each affected enthesis out of the six bilateral sites was scored in terms of inflammatory and morphological components according to the OMERACT enthesitis definition and composite semi-quantitative scale (0–3) (Supplementary Table S2). Two definitions of activity at site level were used to derive two OMERACT (PDUS) enthesitis scores (at patient level) for the first time in this study. Definition 1 combines the rating of inflammatory abnormalities with B-mode (range 0–1) and inflammatory activity with PD signal (range 0–3); Definition 2 uses the PD signal rating only (range 0–3) [30]. The global OMERACT enthesitis score comprises the sum of each single abnormal site of the six bilateral targeted entheses, with a range of 0–48 using Definition 1 and a range of 0–36 using Definition 2. Supplementary Tables S2 and S3 describe the development of the OMERACT enthesitis definition, including the choice of the elementary lesions. Severity was graded with the help of an atlas, available in each center that had examples of B-mode and PD signal grading for each examined enthesis site. For the first time, the definition of activity at anatomical site level was summed up for all sites to derive an OMERACT (PDUS) enthesitis score at patient level by combining the rating of abnormalities detected with B-mode (range 0–1) and PD signal (range 0–3) (Supplementary Table S3).

### Clinical assessments

Clinical assessments included tender and swollen joint counts (TJC and SJC, respectively); clinical enthesitis by SPARCC index, functional disability by Health Assessment Questionnaire Disability Index (HAQ-DI), and evaluation of patient and physician global assessment of disease activity by visual analog scale (VAS) [17].

### Analysis

#### Clustering analysis

Multiple factor analysis (MFA) [31], an extension of principal component analysis that assigns weight to variables and thereby balances the influence of groups of similar variables on the analysis, was performed on the baseline B-mode and PD signal core components of the composite PDUS at joint level. Six blocks of active variables, that is, variables that contribute to the construction of the dimensions and 11 supplementary blocks of variables, were used for the MFA (Supplementary Table S4).

The active blocks correspond to the location and the type of assessment (B-mode and PD signal). In total, 48 baseline active variables were used to perform the MFA analysis using the worst paired signal at joint level for B-mode and PD signal. The additional supplementary blocks correspond to SJC/TJC, clinical enthesitis or composite PDUS by location. Missing data for continuous and categorical variables were imputed through regularized iterative principal components [32]. The number of components leading to the smallest mean squared error of prediction was retained with k-fold cross-validation. One-thousand repeats and the 95% confidence interval (CI) of the bootstrapped eigenvalues distribution were used to identify the number of MFA components to retain for clustering.

To define the number of clusters, Hierarchical Clustering on Principal Components [33] was performed with the Ward’s criterion and an Euclidian distance metric on the selected MFA principal components. The inter-cluster inertia gain was used to select the optimal level of division.

Demographics, clinical, and ultrasound-detected synovitis and enthesitis data were summarized descriptively by cluster using the patient population enrolled in ULTIMATE. Categorical data were presented as frequencies and percentages and continuous data were presented as mean and standard deviation.

#### Longitudinal analysis

Longitudinal analysis of responses in GLOESS over time to secukinumab vs. placebo across the identified clusters were compared with a linear mixed-effects model with random intercept during the first 12 weeks (i.e., placebo vs. secukinumab double-blind evaluation period). The model was adjusted with treatment, center and analysis visit as factors, and weight and baseline GLOESS as continuous covariates. Cluster by treatment by analysis visit was included as an interaction term in the model. A compound symmetry structure was assumed for this model. Adjusted mean changes from baseline are presented comparing secukinumab with placebo at the different scheduled timepoint visits and clusters. 

#### Software

All statistical computations were performed in R version 4.1.0 (2021-05-18), using RStudio version 1.4.1717-3 [34], environment [35]. MFA and Hierarchical Clustering on Principal Components were performed with the factoMineR [36], package in R. Missing data were imputed with the missMDA package in R [32]. Since the cluster analysis was applied to a preexisting data set that was not powered to detect statistically significant differences in treatment response, the differences across cluster are reported as numerical changes.

## Results

The cluster analysis was conducted on all 166 patients enrolled in the ULTIMATE trial. Patients had active disease at baseline with a mean number of 14 tender joints, nine swollen joints, and four clinically active enthesitis. The mean Disease Activity Index for Psoriatic Arthritis (DAPSA) score was similar in both treatment and placebo groups; 34.2 in the secukinumab group and 33.3 in the placebo group (data not reported in previous publication).

A total of three clusters were identified according to their baseline severity of PDUS-detected synovitis after MFA and clustering (Fig. S2A-E and Fig. S3A-B), with 49 (30%) patients assigned to cluster 1, 54 (33%) to cluster 2, and 63 (38%) to cluster 3 (Table 1).

**Table 1:** Baseline characteristics of the patient clusters identified with analysis performed on the baseline B-mode and PD signal core components of the composite PDUS at joint level

Characteristics, mean (SD) unless otherwise specified	Cluster 1 (*n* = 49)	Cluster 2 (*n* = 54)	Cluster 3 (*n* = 63)	Total (*N* = 166)
Demographic characteristics				
Age, years	47 (11)	50 (13)	44 (12)	47 (12)
Male, n (%)	19 (39)	28 (52)	28 (44)	75 (45)
Time since diagnosis of PsA, years	6.9 (6.3)	7.2 (7.9)	5.3 (6.3)	6.4 (6.9)
Current smokers, n (%)	10 (20)	6 (11)	8 (13)	24 (14)
Body weight, kg	78 (18)	85 (20)	80 (18)	81 (19)
BMI, kg/m^2^	28 (7)	29 (5)	29 (6)	29 (6)
Clinical characteristics				
Tender joint count (out of 78 joints)	12 (8)	17 (13)	12 (8)	14 (10)
Swollen joint count (out of 76 joints)	8.1 (7)	12 (11)	8 (5)	9 (8)
SPARCC enthesitis index	3.8 (3.5)	4.3 (3.1)	4.6 (2.9)	4.3 (3.1)
Patient assessment of psoriatic arthritis pain (VAS)	60 (22)	63 (20)	54 (24)	59 (23)
Patient global assessment of disease activity (VAS)	59 (21)	62 (21)	58 (26)	60 (23)
Physician global assessment of disease activity (VAS)	52 (14)	56 (22)	54 (22)	54 (20)
HAQ-DI score	1.2 (0.6)	1.3 (0.7)	1.2 (0.7)	1.2 (0.7)
High-sensitivity C-reactive protein, mg/L	11 (16)	13 (18)	10 (13)	11 (16)
CASPAR Score	4.0 (0.9)	4.4 (1.0)	4.5 (0.8)	4.3 (0.9)
Psoriasis, n (%)	22 (45)	28 (52)	19 (30)	69 (42)
PASI score*	9.3 (6.9)	9.9 (7.9)	9.9 (8.0)	9.7 (7.5)
Ultrasound characteristics				
Number of joints with synovitis detected by ultrasound	4 (2)	13 (5)	11 (4)	10 (5)
GLOESS PDUS score	10 (6)	35 (18)	30 (11)	26 (16)
GLOESS synovial hypertrophy (B-mode)	10 (6)	34 (18)	29 (11)	25 (16)
GLOESS PD signal	5 (3)	13 (10)	5 (4)	7 (7)
OMERACT enthesitis score (definition 1)	4.8 (3.8)	6.5 (4.7)	5.8 (3.7)	5.8 (4.1)

Global OMERACT (PDUS) enthesitis score (definition 1) ranges from 0 to 48 and is the sum of the B-mode (0 = absence, 1 = presence) and PD signal across 12 enthesitis sites

*CASPAR* ClASsification criteria for Psoriatic Arthritis, *GLOESS* Global EULAR-OMERACT synovitis score, *HAQ-DI* Health Assessment Questionnaire-Disability Index *OMERACT*, Outcome Measures in Rheumatology *PASI* Psoriasis Area and Severity Index *PD* Power Doppler, *PDUS* Power Doppler ultrasound, S*PARCC* Spondyloarthritis Research Consortium of Canada, *VAS* Visual analog scale (range 0–100)

*Only for patients with psoriasis

### Baseline characteristics of the ultrasound clusters

The overall baseline demographics as well as the clinical and ultrasound characteristics have been reported previously [17]. The baseline demographics were balanced across the three clusters.

Higher GLOESS scores were observed in clusters 2 (indicating the most severe PDUS synovitis) and 3 (indicating an intermediate level of PDUS synovitis) compared with cluster 1 (indicating a mild level of PDUS synovitis). The clinical characteristics showed higher TJC and SJC scores in cluster 2 than in clusters 1 and 3 and similar clinical SPARCC enthesitis index scores and OMERACT enthesitis scores in all clusters **(**Table 1**)**.

**Fig. 1:**
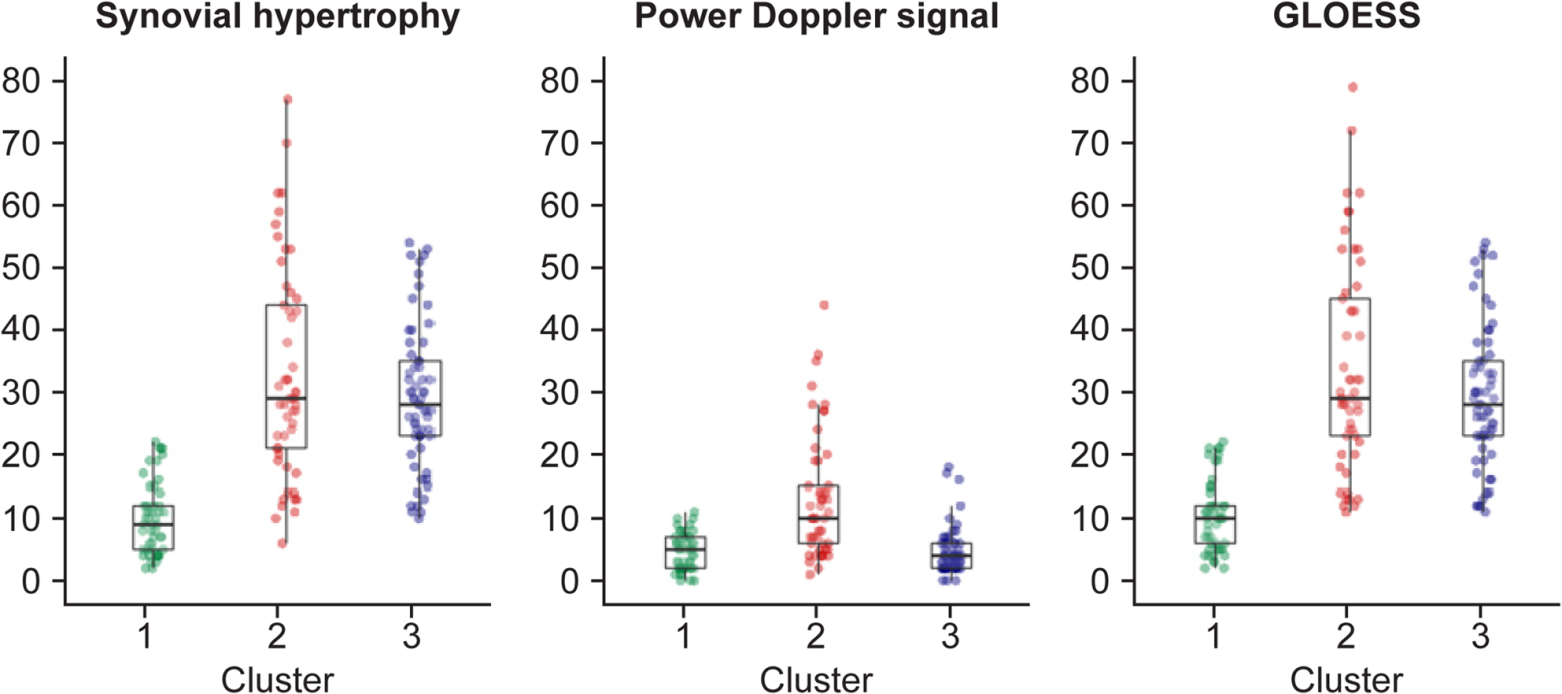
Boxplot of B-mode and PD signal core components and composite PDUS for each cluster identified using post-hoc cluster analysis. GLOESS, global OMERACT-EULAR synovitis score; PD, power Doppler; PDUS, power Doppler ultrasound

**Fig. 2:**
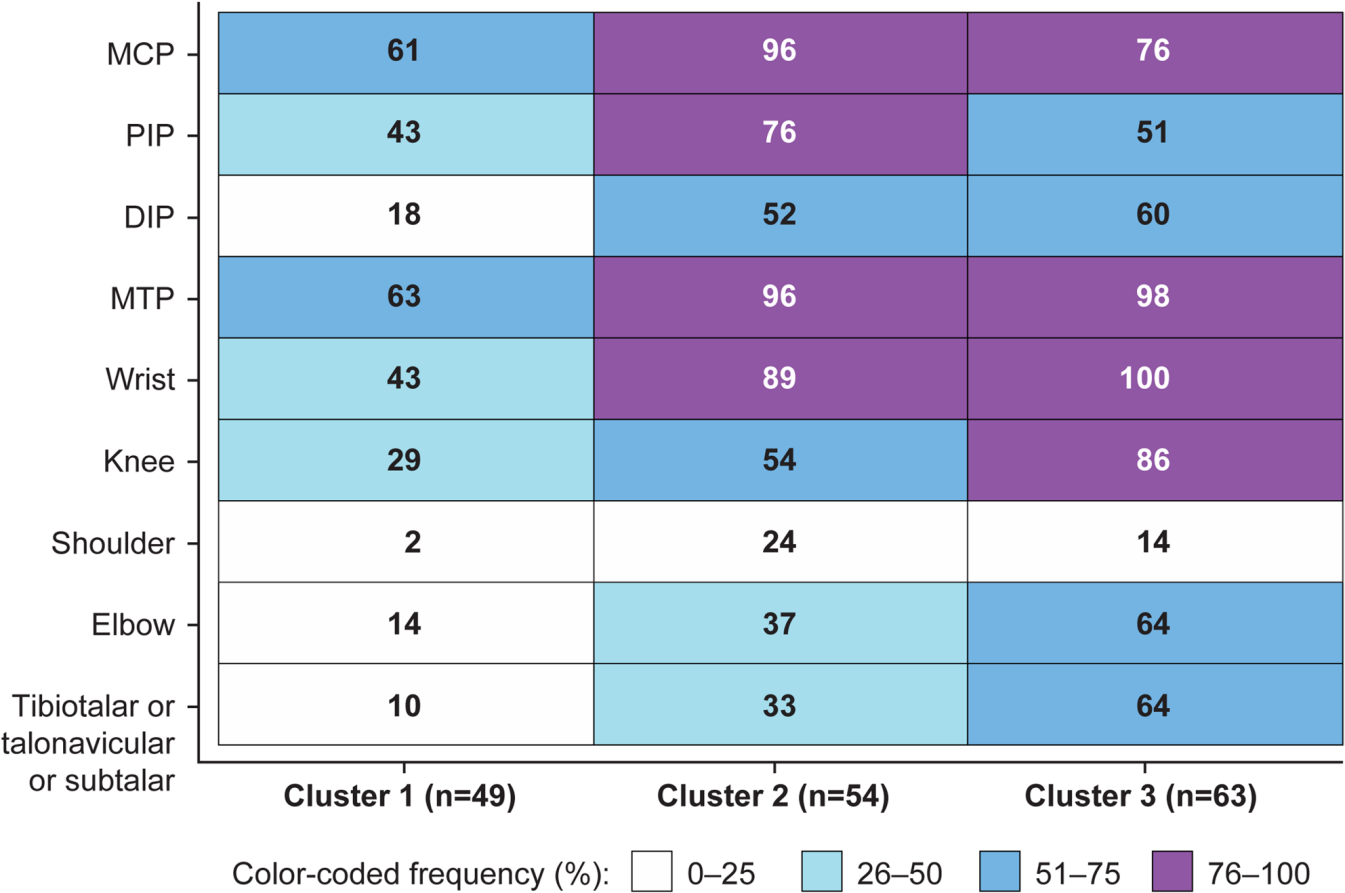
Heat map reporting the location of ultrasound-detected synovitis for each cluster identified using post-hoc analysis. Box color and value represent frequency of identified synovitis in the different joints. At least one joint positive by PDUS for synovitis: MCP 1–5, MTP 1–5, DIP 2–5, PIP 1–5. DIP, distal interphalangeal joint; MCP, metacarpophalangeal joint; MTP, metatarsophalangeal joint; PDUS, power Doppler ultrasound; PIP 1–5, proximal interphalangeal joints 1–5

The mean B-mode core components of GLOESS were higher in clusters 2 and 3 than in cluster 1 (with the value for cluster 2 being slightly higher than that for cluster 3), and the mean PD signal core component of GLOESS was higher in cluster 2 than clusters 1 and 3 (Table 1; Fig. 1). Differences in the joint distribution of PDUS-detected synovitis across the three clusters are shown in the heatmap (Fig. 2). Clusters 2 and 3 had more active synovitis in small and large joints than cluster 1. PDUS-detected synovitis most frequently (≥ 70%) affected MTP, MCP, and wrist joints in clusters 2 and 3 compared with cluster 1. In contrast, cluster 3 had a higher frequency of PDUS synovitis in elbow, knee, and ankle joints compared with cluster 2 (Fig. 2). More detailed description of the baseline joint distribution of PDUS synovitis in the three clusters can be found in Supplementary Table S5.

### Response to treatment at patient level and joint level

**Fig. 3:**
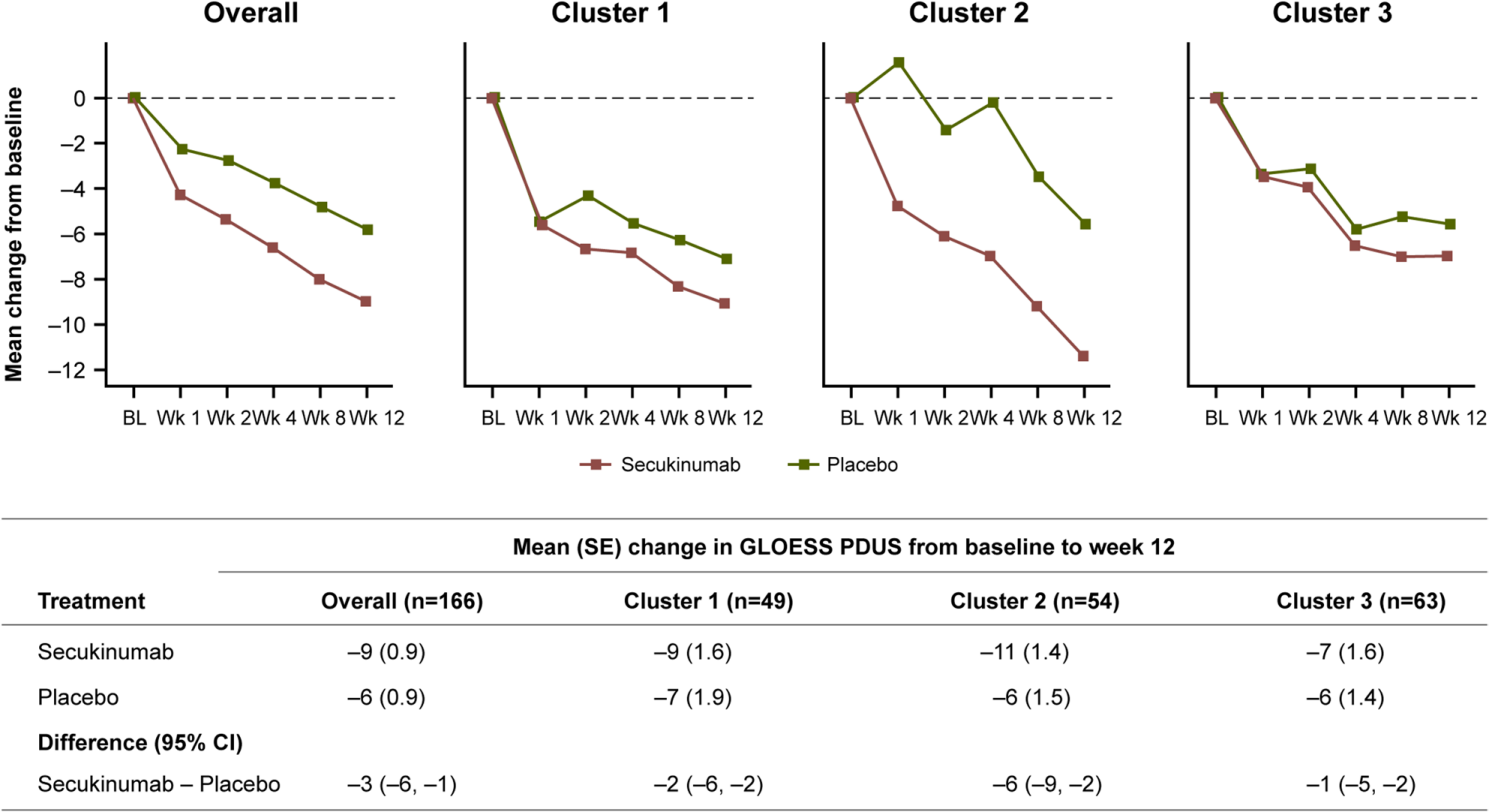
Change in GLOESS from baseline to week 12 by ultrasound cluster (identified by post-hoc analysis on baseline B-mode and PD signal core components of the composite PDUS at joint level) and treatment. BL, baseline; GLOESS, global OMERACT-EULAR synovitis score; PDUS, power Doppler ultrasound; Wk, week

The longitudinal trajectories of the three clusters for GLOESS from baseline to week 12 showed a numerical trend toward a treatment difference favoring secukinumab compared with placebo in all three clusters (Fig. 3). Both core components of SH (B-mode) and PD signal showed treatment differences for secukinumab vs. placebo in cluster 2, in contrast to clusters 1 and 3 (Supplementary Table S6).

**Fig. 4:**
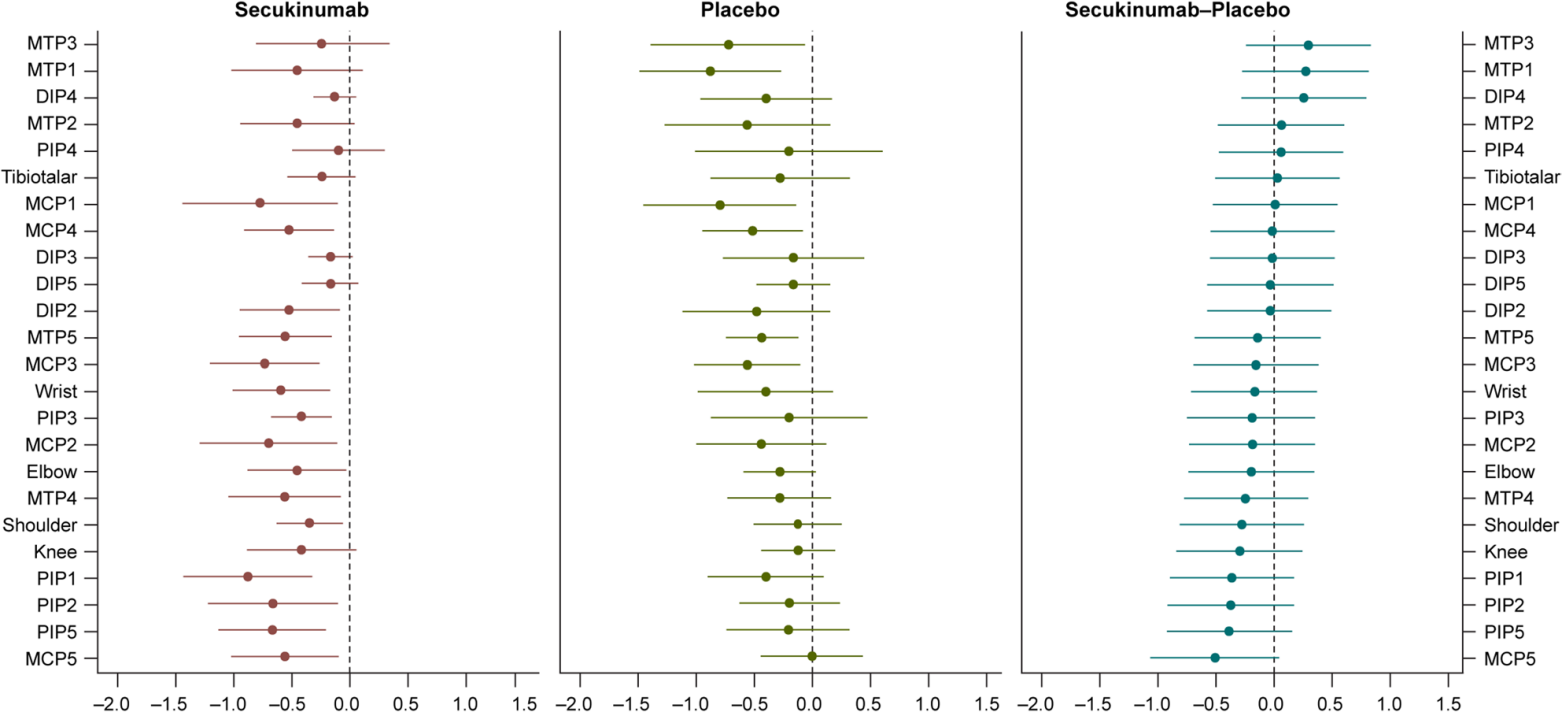
Change from baseline to week 12 in PDUS-detected synovitis by treatment arm in cluster 2 (identified by post-hoc analysis on baseline B-mode and PD signal core components of the composite PDUS at joint level). MCP, metacarpophalangeal; MTP, metatarsophalangeal; PIP, proximal interphalangeal

Differences in response at joint and enthesitis level were also observed in the three clusters. Cluster 2 represented patients with a relatively high score and severe ultrasound-detected synovitis and enthesitis at baseline. In cluster 2, higher responses, mainly on MCP, MTP, wrist, knee and elbow joints, were shown in the secukinumab treatment group compared with the placebo group at week 12 (Fig. 4). Lateral epicondyle, quadriceps tendon insertion, and patellar ligament insertion were the most frequent ultrasound-detected enthesitis at baseline, and these entheseal sites also showed greater improvement with secukinumab vs. placebo at week 12 (Supplementary Fig. S4).

Cluster 3 was characterized by a predominance of moderate to severe synovitis on MTP, wrists, elbows, and knees, which displayed good response to secukinumab compared with placebo, whereas small joints from the hands were less impacted (Supplementary Fig. S5). Cluster 3 presented with a similar distribution of ultrasound enthesitis to cluster 2, with mild severity at baseline and lower response to secukinumab at week 12 (Supplementary Fig. S4).

Cluster 1 presented with a similar severity of synovitis to clusters 2 and 3, with a lower GLOESS and a lower response to secukinumab vs. placebo at 12 weeks (Supplementary Fig. S5). Distribution of enthesitis also overlapped with clusters 2 and 3 with additional moderate to severe Achilles enthesitis, but the level of severity across the different enthesitis sites was moderate to severe and a good response to secukinumab vs. placebo on these sites was observed at week 12 (Supplementary Fig. S4).

## Discussion

This is the first study in patients with active PsA in which clustering utilizing ML-based methods on ultrasonographic synovitis data were performed and analyzed. The three clusters identified were mainly differentiated by the level of inflammation based on PD signal, and heterogeneity of joint distribution.

Cluster 2 represented the most affected group of patients, characterized by the highest score of active PDUS synovitis, mainly driven by PD signal, a higher number of involved joints, and higher TJC and SJC. In contrast, patients in cluster 3 had less severe synovitis in terms of PD signal and slightly lower frequency of location of joints involved. Interestingly, PDUS synovitis in cluster 3 was mainly detected in the large joints (e.g., elbow, knee, and ankle) and involved a lower frequency of MCP and PIP joints compared with patients in cluster 2. This difference in PD activity and distribution of joints involved may suggest either a different origin of synovitis (e.g., more osteoarthritic synovitis) or difficulty in detecting PD signal in large joints (i.e., lower sensitivity of PD for deeper joints). However, it could also reflect a different pattern of PsA with a different therapeutic response or a different time to treatment response (e.g., knee and ankle joints are weight-bearing joints and effective treatment response might take longer compared with non–weight-bearing joints). As in all therapeutic trials, additional confounding mechanical causes of synovitis were not investigated. Indeed, joints were not evaluated for concomitant osteoarthritis (OA) features, which would have provided additional information to differentiate the origin of joint inflammation (i.e., active PsA vs. OA). Moreover, the different distribution of joints involved (large deep joints vs. small superficial joints) may also explain the observed higher prevalence of B-mode synovial hypertrophy than PD signal in cluster 3.

Although the cluster analysis sample was not powered to allow detection of statistically significant differences in treatment response (i.e., p-value supported), the analysis of the longitudinal trajectory of secukinumab and placebo revealed differences in the treatment response, across the clusters, over the first 12-week period. Change in GLOESS from baseline to week 12 for all three clusters showed numerical treatment differences favoring secukinumab vs. placebo; a larger difference in GLOESS and each core component (SH and PD signal) was only observed in favor of secukinumab in cluster 2 in contrast with clusters 1 and 3. The better treatment response with secukinumab in patients from cluster 2 may be related to a higher inflammatory burden with more severe and extensive synovitis. In contrast, patients from cluster 1 at baseline presented with a low active disease on synovitis as shown by both a lower GLOESS and its core components and a lower number of synovitis than clusters 2 and 3, which may explain the lack of difference in treatment response together with the small sample size. In cluster 1, it is striking to observe a disconnect between the number of swollen joints and the number of PDUS-detected synovitis.

All patients enrolled in the ULTIMATE trial met the clinical and ultrasound entry criteria of activity and had clinically defined PsA (polyarthritis defined by at least three clinically tender joints and three clinically swollen joints), highlighting the heterogeneity within this patient population and the importance of identifying disease sub-phenotypes [17].

PDUS allows objective visualization of the activity and severity of synovitis to complement clinical assessment of swollen joints and helps in evaluating inter-investigator variability and natural fluctuation of the inflammation. The patterns of PDUS severity across the three clusters may have influenced the longitudinal trajectory of the response, and the lack of difference between secukinumab and placebo in two clusters. The results of this analysis may be useful in clinical practice because they highlight the heterogeneity of distribution and severity of synovitis in PsA and also emphasize the utility of ultrasound in complementing clinical assessment (i.e., clinically and imaging driven objective assessment of treatment response).

### Study limitations

Some limitations should be acknowledged. These findings were only evaluated in the ULTIMATE patient population with a limited sample size. To evaluate the clinical utility of these clusters, external validation in other datasets is needed. The choice of the variables used for the MFA groups, the number of components retained to perform the clustering as well as the determination of the optimal number of clusters may have an influence on the attribution of patients in a specific cluster. Moreover, there may be a disconnect between clinical improvement and the level of inflammation recorded by PDUS at joint level. In addition, we did not integrate other ultrasound variables to characterize patients, such as PDUS enthesitis, because the OMERACT enthesitis scores used in the ULTIMATE study were exploratory and not yet fully validated. Finally, this was an exploratory post-hoc analysis with data summarized descriptively and was not powered to evaluate the difference between treatment with secukinumab vs. placebo across the clusters. We acknowledge the limitation that no correlation with plain films or analysis of changes in OA was undertaken in this study to support differences in treatment response based on differences in the pathogenesis of inflammation and joint distribution.

## Conclusion

This exploratory cluster analysis on the ULTIMATE study highlights the heterogeneity of a typical PsA trial population in terms of severity of synovitis and the number and distribution of affected joints. These differences may explain the difference in the trajectory of treatment response. A combined clinical and PDUS approach is in line with EULAR and GRAPPA recommendations and should be considered when designing future PsA therapeutic trials, to ensure a more homogeneous patient population in terms of severity of synovitis, to optimize treatment response.

## Supplementary Information

Supplementary Material 1.

## Data Availability

All data supporting the findings of this study are available within the paper and its Supplementary Information.

## Abbreviations

ACR: American College of Rheumatology
CASPAR: ClASsification criteria for Psoriatic Arthritis
DAPSA: Disease Activity Index for Psoriatic Arthritis
DMARD: Disease-modifying antirheumatic drug
DIP: Distal interphalangeal joint
EULAR: European Alliance of Associations for Rheumatology
GLOESS: Global OMERACT-EULAR Synovitis Score
GRAPPA: Group for Research and Assessment of Psoriasis and Psoriatic Arthritis
HAQ-DI: Health Assessment Questionnaire Disability Index
NSAID: Nonsteroidal anti-inflammatory drug
MCP: Metacarpophalangeal joint
MFA: Multiple factor analysis
ML: Machine learning
MTP: Metatarsophalangeal joint
OMERACT: Outcome Measures in Rheumatoid Arthritis Clinical Trials
PD: Power Doppler
PDUS: Power Doppler ultrasound
PIP: Proximal interphalangeal joint
PsA: Psoriatic arthritis
SH: Synovial hypertrophy
SJC: Swollen joint count
SpA: Spondyloarthritis
SPARCC: Spondyloarthritis Research Consortium of Canada
TJC: Tender joint count
US: Ultrasound
VAS: Visual Analog Scale
