# Supplementary material for: Exploring clusters based on ultrasound-detected inflammation in patients with psoriatic arthritis: a post-hoc analysis from the ULTIMATE trial
Source: BMC Musculoskelet Disord. 2026 Jan 30;27:106. doi: 10.1186/s12891-025-09434-w (PMC12874723; doi:10.1186/s12891-025-09434-w)
Supplement: Supplementary file 1 — Supplementary Material 1. [file 12891_2025_9434_MOESM1_ESM.docx]

**Supplementary Appendix**

#

**Exploring clusters based on ultrasound-detected inflammation in patients with psoriatic arthritis: a post-hoc analysis from the ULTIMATE trial**

**Authors:** Maria Antonietta D’Agostino, Philip G Conaghan, Corine Gaillez, Maarten Boers, Esperanza Naredo, Peter Mandl, Philippe Carron, Alejandra López-Rdz, Ruben Burgos-Vargas, Javier Rosa, Catherine Bakewell, Tomas Cazenave, Weibin Bao, David Demanse, Georg Schett

# Table of contents

| **Supplementary item** | **Page** |
| --- | --- |
| Study inclusion and exclusion criteria | [2](#_Study_inclusion_and) |
| Multiple factor analysis and clustering | [8](#_Multiple_factor_analysis) |
| Supplementary Figures | [10](#_Supplementary_Figures) |
| Fig. S1 | 10 |
| Fig. S2 | [12](#_Supplementary_Figure_Fig._1) |
| Fig. S3 | [18](#_Supplementary_Figure_S3.) |
| Fig. S4 | [20](#_Supplementary_Fig._S4.) |
| Fig. S5 | [22](#_Supplementary_Fig._S5.) |
| Supplementary Tables | [24](#_Supplementary_Fig._S5.) |
| Table S1 | [24](#_Supplementary_Table_S1.) |
| Table S2 | [25](#_Supplementary_Table_S2.) |
| Table S3 | [26](#_Supplementary_Table_S3.) |
| Table S4 | [27](#_Supplementary_Table_S4) |
| Table S5 | [35](#_Supplementary_Table_S5.) |
| Table S6 | [36](#_Supplementary_Table_S6.) |

# Study inclusion and exclusion criteria

**Inclusion criteria**

1. Patient must be able to understand and communicate with the Investigator and comply with the requirements of the study and must provide written, signed, and dated informed consent before any study assessment is performed.
2. Male or female patients at least 18 years of age.
3. Diagnosis of PsA as per CASPAR with active PsA for at least 6 months and a TJC ≥3 of 78 joints and SJC ≥3 of 76 joints at baseline.
4. a) Patients must have a total synovitis PDUS score ≥2 and inflammation related to PD signal ≥2 for at least 1 affected joint (as observed via PDUS) of 48 joints at the screening visit and at the baseline visit (before injection), OR

b) Patients must have a total synovitis PDUS score ≥2 and inflammation related to PD signal ≥1 for at least 2 affected joints (as observed via PDUS) of 48 joints at the screening visit and at the baseline visit (before injection).

1. At least 1 clinically involved enthesitis site at screening and at the baseline visit (before injection) defined by Spondyloarthritis Research Consortium of Canada (SPARCC) index different from 0.
2. Rheumatoid factor and anti-cyclic citrullinated peptide (anti-CCP) negative at screening.
3. Patients with PsA who have an inadequate response to non-biologic disease-modifying anti-rheumatic drugs (DMARDs).
4. Patients with PsA who are taking NSAIDs should be on a stable dose for at least 2 weeks prior to enrollment and remain on a stable dose throughout the 24-week study period unless rescue therapy is needed.
5. Patients with PsA who are taking steroids must have received steroids at least 3 months prior to the baseline visit and be on a stable dose of ≤10-mg equivalent prednisone for at least 4 weeks prior to baseline visit and remain on a stable dose throughout the 24-week study period unless tolerance issues are present.
6. Patients with PsA who are taking methotrexate (MTX) or DMARDs must have received them at least 3 months prior to baseline visit and be on a stable dose of ≤25 mg/week of MTX or stable standard doses of other DMARDs (according to the Investigator’s judgement) for at least 4 weeks prior to the baseline visit and remain on a stable dose throughout the 24-week study period.

**Exclusion criteria**

1. Chest X-ray or chest MRI with evidence of ongoing infectious or malignant process obtained within 3 months prior to screening and evaluated by a qualified physician.
2. Previous exposure to secukinumab or other biologic drugs directly targeting interleukin (IL)-17 or IL-17 receptor.
3. Patients taking high-potency opioid analgesics (e.g., methadone, hydromorphone, morphine).
4. Use of any investigational drug and/or devices within 4 weeks before randomization or a period of 5 half-lives of the investigational drug, whichever is longer.
5. Any change in the dose of oral corticosteroids in the last 4 weeks prior to the baseline visit or use of i.v. intra-muscular or intra-articular corticosteroid during the last 4 weeks prior to the enrollment visit.
6. Patients who have previously been treated with tumor necrosis factor (TNF) α inhibitors (investigational or approved).
7. History of hypersensitivity to the study drug or its excipients or to drugs of similar classes.
8. Previous treatment with any cell-depleting therapies including but not limited to anti-CD20 investigational agents (e.g., CAMPATH, anti-CD4, anti-CD5, anti-CD3, anti-CD19).
9. Prohibited psoriasis treatments/medications with topical corticosteroids in the last 4 weeks prior to randomization.
10. Pregnant or nursing (lactating) women, where pregnancy is defined as the state of a female after conception and until the termination of gestation, confirmed by a positive human chorionic gonadotropin (hCG) laboratory test.
11. Women of childbearing potential, defined as all women physiologically capable of becoming pregnant, unless they are using effective methods of contraception during the entire study or longer if required by locally approved prescribing information (e.g., 20 weeks in EU). Effective contraception methods include:

- Total abstinence (when this is in line with the preferred and usual lifestyle of the patient. Periodic abstinence (e.g., calendar, ovulation, symptothermal, post-ovulation methods) and withdrawal are not acceptable methods of contraception.
- Female sterilization (have had surgical bilateral oophorectomy with or without hysterectomy) or tubal ligation at least 6 weeks before taking study drug. In case of oophorectomy alone, only when the reproductive status of the woman has been confirmed by follow-up hormone-level assessment.
- Male sterilization (at least 6 months prior to screening). For female patients on the study, the vasectomized male partner should be the sole partner for that patient.
- Barrier methods of contraception: condom or occlusive cap (diaphragm or cervical/vault caps) with spermicidal foam/gel/film/cream/vaginal suppository.
- Use of oral, injected, or implanted hormonal methods of contraception or other forms of hormonal contraception that have comparable efficacy (failure rate < 1%), for example, hormone vaginal ring or transdermal hormone contraception.
- Placement of an intrauterine device or intrauterine system.
- In case of use of oral contraception, women should have been stable on the same pill for a minimum of 3 months before taking study drug. Women are considered post-menopausal and not of childbearing potential if they have had 12 months of natural (spontaneous) amenorrhea with an appropriate clinical profile (e.g., age appropriate, history of vasomotor symptoms) or surgical bilateral oophorectomy (with or without hysterectomy) or tubal ligation at least 6 weeks ago. In the case of oophorectomy alone, only when the reproductive status of the woman has been confirmed by follow-up hormone level assessment is, she considered not of childbearing potential.

1. Active ongoing inflammatory diseases other than PsA that might confound the evaluation of the benefit of secukinumab therapy.
2. Underlying metabolic, hematologic, renal, hepatic, pulmonary, neurologic, endocrine, cardiac, infectious, or gastrointestinal conditions which in the opinion of the investigator immunocompromise the patient and/or place the patient at unacceptable risk for participation in an immunomodulatory therapy.
3. Significant medical problems or diseases, including but not limited to the following: uncontrolled hypertension (≥ 160/95 mmHg), congestive heart failure (New York Heart Association status of class III or IV), and uncontrolled diabetes (as per investigator’s judgment).
4. History of clinically significant liver disease or liver injury as indicated by abnormal liver function tests (LFT) such as aspartate aminotransferase (AST), alanine aminotransferase (ALT), alkaline phosphatase, or serum bilirubin. The investigator should be guided by the following criteria:
   1. Any single parameter may not exceed 2 x upper limit of normal (ULN). A single parameter elevated up to and including 2 x ULN should be re-checked once more as soon as possible, and in all cases, at least prior to enrollment/randomization, to rule out laboratory error.
   2. If the total bilirubin concentration is increased above 2 x ULN, total bilirubin should be differentiated into the direct and indirect reacting bilirubin. In any case, serum bilirubin should not exceed 1.6 mg/dL (27 μmol/L).
5. History of renal trauma, glomerulonephritis, or patients with 1 kidney only, or a serum creatinine level exceeding 1.5 mg/dL (132.6 μmol/L).
6. Screening total white blood cell (WBC) count <3000/μL, or platelets <100,000/μL or neutrophils <1500/μL or hemoglobin < 8.5 g/dL (85 g/L).
7. Active systemic infections during the last 2 weeks (exception: common cold) prior to randomization.
8. History of ongoing, chronic, or recurrent infectious disease or evidence of tuberculosis infection as defined by either a positive purified protein derivative (PPD) skin test (the size of induration will be measured after 48-72 hours, and a positive result is defined as an induration of ≥5 mm or according to local practice/guidelines) or a positive QuantiFERON TB-Gold test.
9. Patients with a positive test may participate in the study if further workup (according to local practice/guidelines) establishes conclusively that the patient has no evidence of active tuberculosis. If presence of latent tuberculosis is established, then treatment according to local country guidelines must have been initiated.
10. Known infection with human immunodeficiency virus, hepatitis B, or hepatitis C at screening or randomization.
11. History of lymphoproliferative disease or any known malignancy or history of malignancy of any organ system within the past 5 years (except for basal cell carcinoma or actinic keratoses that have been treated with no evidence of recurrence in the past 3 months, carcinoma *in situ* of the cervix or non-invasive malignant colon polyps that have been removed).
12. Current severe progressive or uncontrolled disease, which in the judgment of the clinical Investigator renders the patient unsuitable for the trial.
13. Inability or unwillingness to undergo repeated venepuncture (e.g., because of poor tolerability or lack of access to veins).
14. Any medical or psychiatric condition which, in the Investigator’s opinion, would preclude the participant from adhering to the protocol or completing the study per protocol.
15. Donation or loss of 400 mL or more of blood within 8 weeks before randomization.
16. History or evidence of ongoing alcohol or drug abuse, within the last 6 months before randomization.
17. Plans for administration of live vaccines during the study period or within 6 weeks preceding randomization.

# Multiple factor analysis and clustering

**Supplementary Figs. S2A-E** visualize the results of multiple factor analysis (MFA) for the first two dimensions. To determine the number of components, the 5^th^ and 95^th^ percentiles of the eigenvalues from the resulting projection of 1000 MFA bootstrap were calculated. Non-overlapping percentiles were used to select the components to retain (**Supplementary Fig. S2A**). Two significant MFA dimensions were retained during the bootstrapping procedure. The proportion of the variance explained by each principal component from the MFA is shown in **Supplementary Fig. SB**. As suggested by the data shown in **Supplementary Fig. S2B**, two components explained 23.4% of the variability and selected for the clustering. While the first dimension was dominated by B-mode (**Supplementary Figs. S2C-E**), the second dimension predominantly contained variables derived from PD-mode in hands. After the Hierarchical Clustering on Principal Components (HCPC) on the two significant MFA dimensions, subjects were grouped in three clusters based on the inter-cluster inertia gains (**Supplementary Fig. S3A**) and projected into the first two components of the MFA (**Supplementary Fig. S3B**). The inertia gains decreased suggesting an optimal number of clusters of 3.

# Supplementary Figures

## Supplementary Fig. S1 Study design

BSA, body surface area; BSL, baseline; DMARD, disease-modifying anti-rheumatic drug; GLOESS, Global OMERACT-EULAR Synovitis Score; IR, inadequate response; IRT, Interactive Response Technology; N, number of randomized patients; NSAIDs, nonsteroidal anti-inflammatory drugs; OMERACT, Outcome Measures in Rheumatoid Arthritis Clinical Trials; PASI, Psoriasis Area and Severity Index; R, randomization; SC, subcutaneous.

**
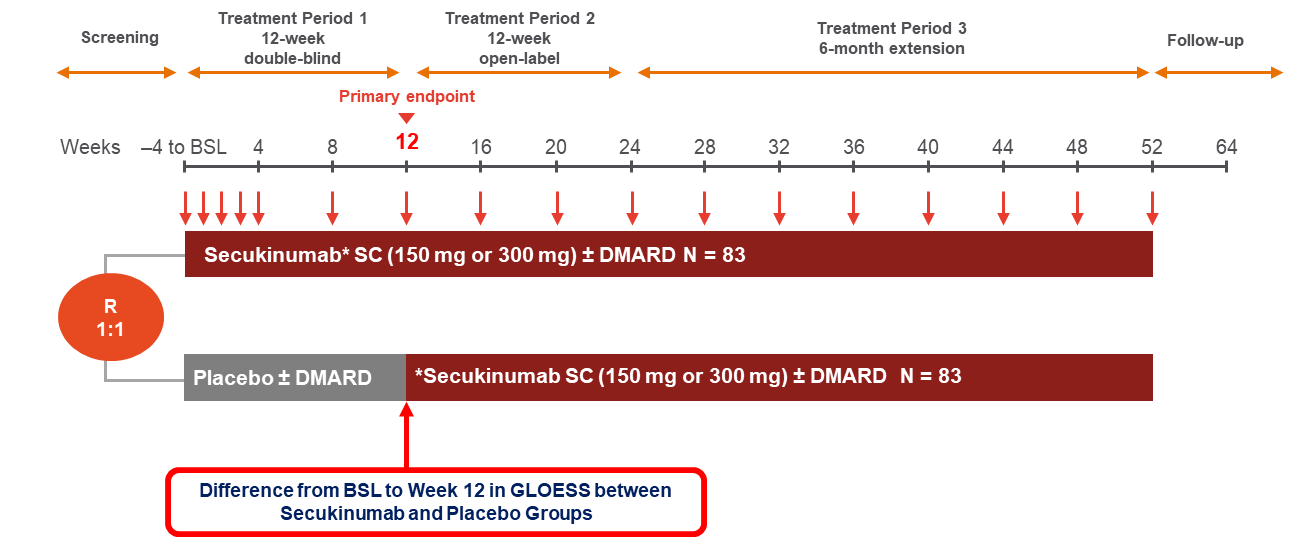
**

*Patients randomized to secukinumab arm received either 300 mg (BSA >10% as assessed by PASI score) or 150 mg (BSA ≤10%) dosing according to the severity of skin psoriasis through IRT. The PASI score was assessed for patients in whom at least 3% of the BSA was affected by psoriatic skin involvement at baseline and subsequent visits. A follow-up visit was performed 12 weeks after the last study treatment administration for all patients, regardless of whether they completed the entire study as planned or discontinued prematurely. Patients could continue to receive NSAIDs at a stable dose for at least 2 weeks prior to enrollment and remain on a stable dose through the first 24-weeks unless rescue therapy is needed. Patients could continue to receive the following medications at a stable standard dose from 1-month prior to screening to 24 weeks: methotrexate (≤25 mg/week), glucocorticoids (≤10 mg/day prednisone or equivalent), and NSAIDs.

Supplementary Fig. S2 Analyses contributing to MFA. A) Bootstrap analysis for number of principal components. B) Percentage of the variance explained by each dimension of the MFA. C) Contribution of the group of variables to the MFA dimensions 1 and 2. The bar graphs represent the weight of the single group of variables on the representativeness of the MFA dimensions 1 and 2 as expressed by the percentage of their contribution. The reference dashed line in red shown on the bar plot corresponds to the expected value if the contribution where uniform. D) Projection of the group of variables in the MFA. The coordinates which indicate the correlation that exists between each group of variables (active in black and supplementary in red) and the factors resulting from the MFA are presented for dimensions 1 and 2. E) Variable projection according to MFA. Dimensions 1 and 2 of the MFA characterize the projections of the categories (or modalities coded as 0 versus 1+) of active variables (points) and group of variables (in colors). All positive categories are positioned on the right of the graph (along the horizontal axis) and on the left for absence of synovitis, which also depicts the influence of B-mode in foot, large joints, and hands. Positive synovitis for PD-mode and B-mode in hand are along the vertical axis located on the top of the figure.
BMODE, B-mode; DMODE, PD-mode; CPDUS, GLOESS PDUS score; MFA, multiple factor analysis; PD, power Doppler.

**A) Bootstrap analysis for number of principal components**


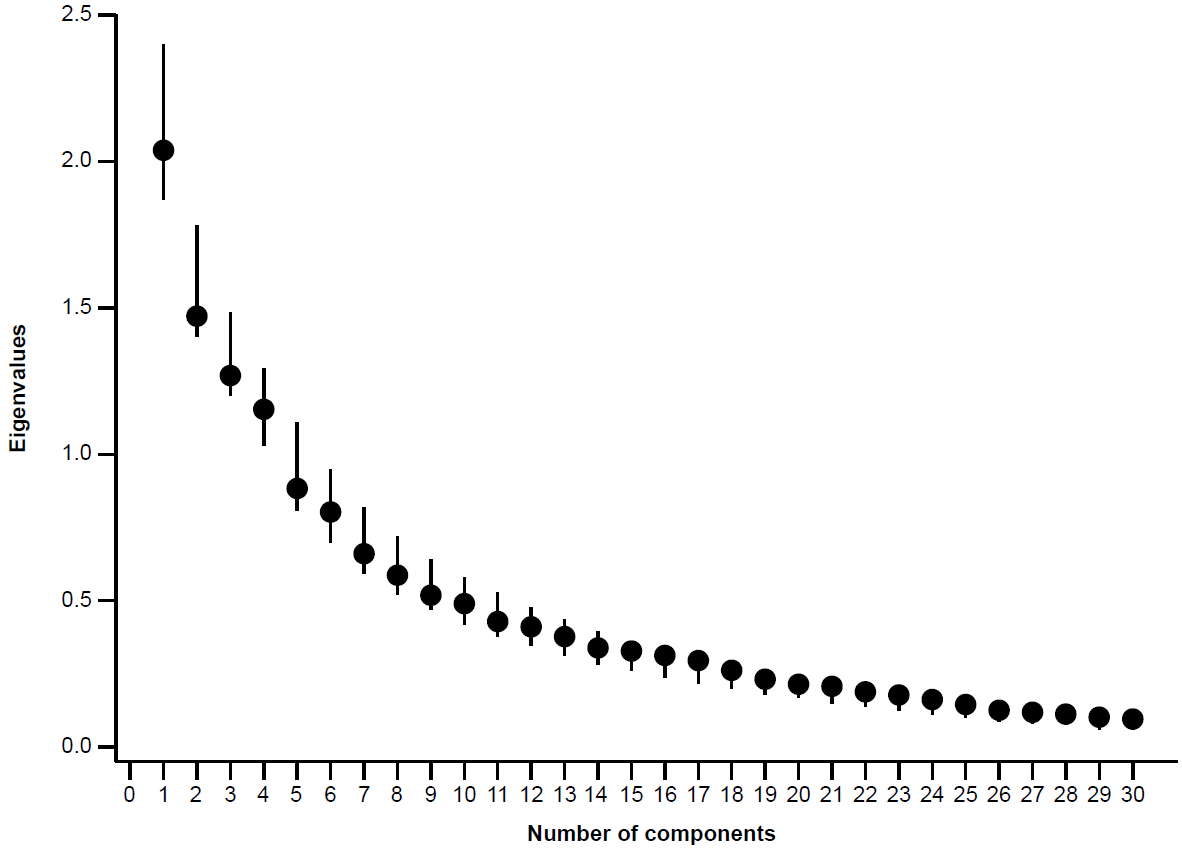


**B) Percentage of the variance explained by each dimension of the MFA**


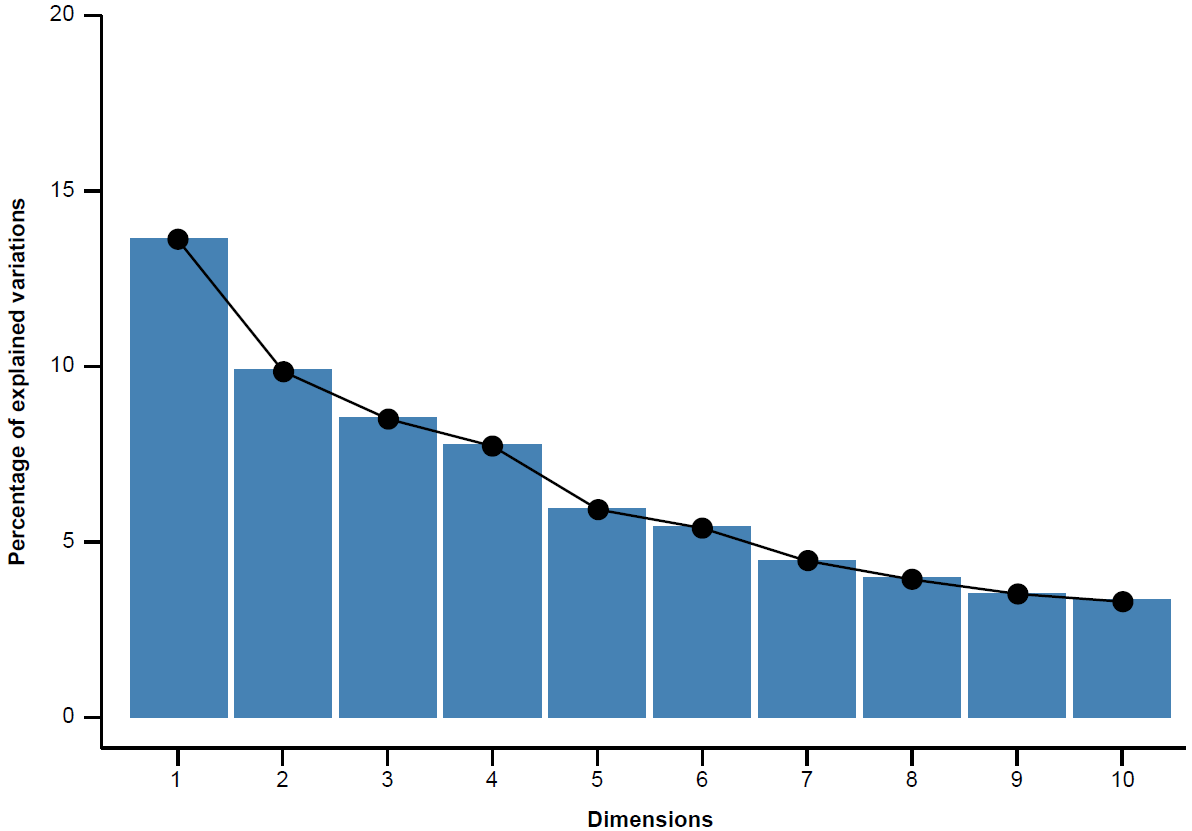


**C) Contribution of the group of variables to the MFA dimensions 1 and 2**


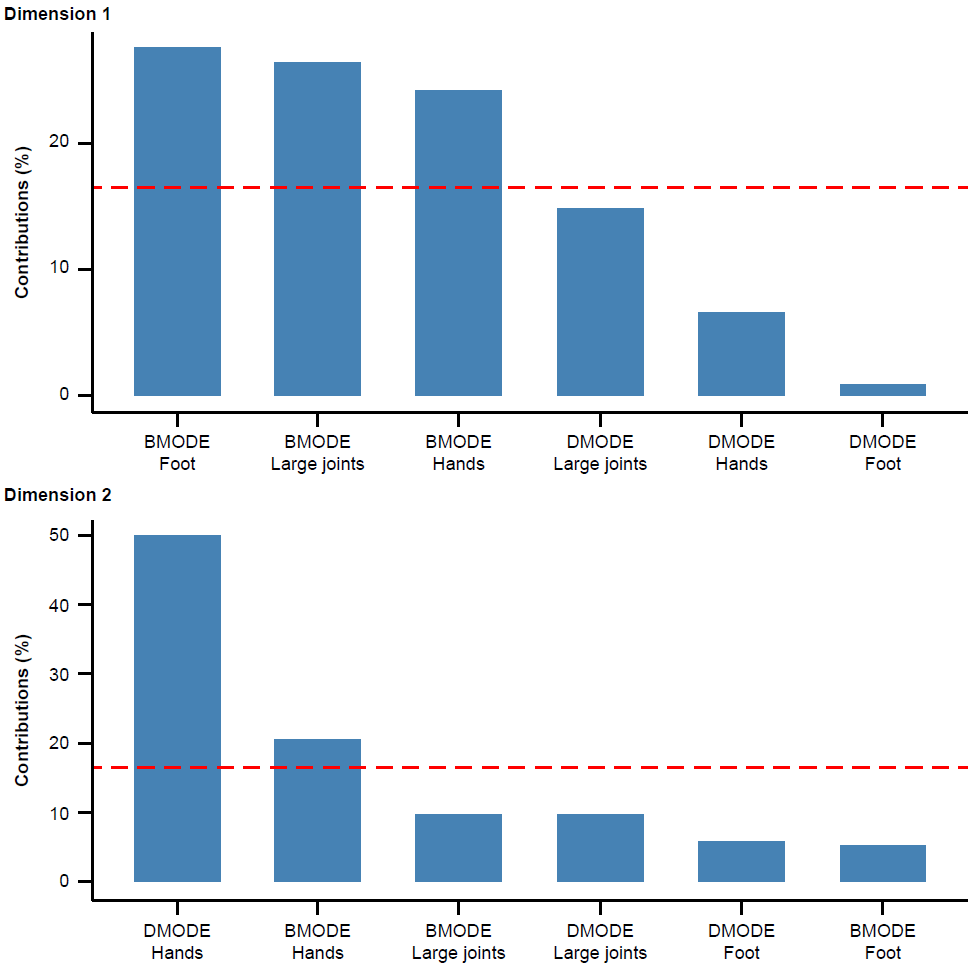


**D) Projection of the group of variables in the MFA**


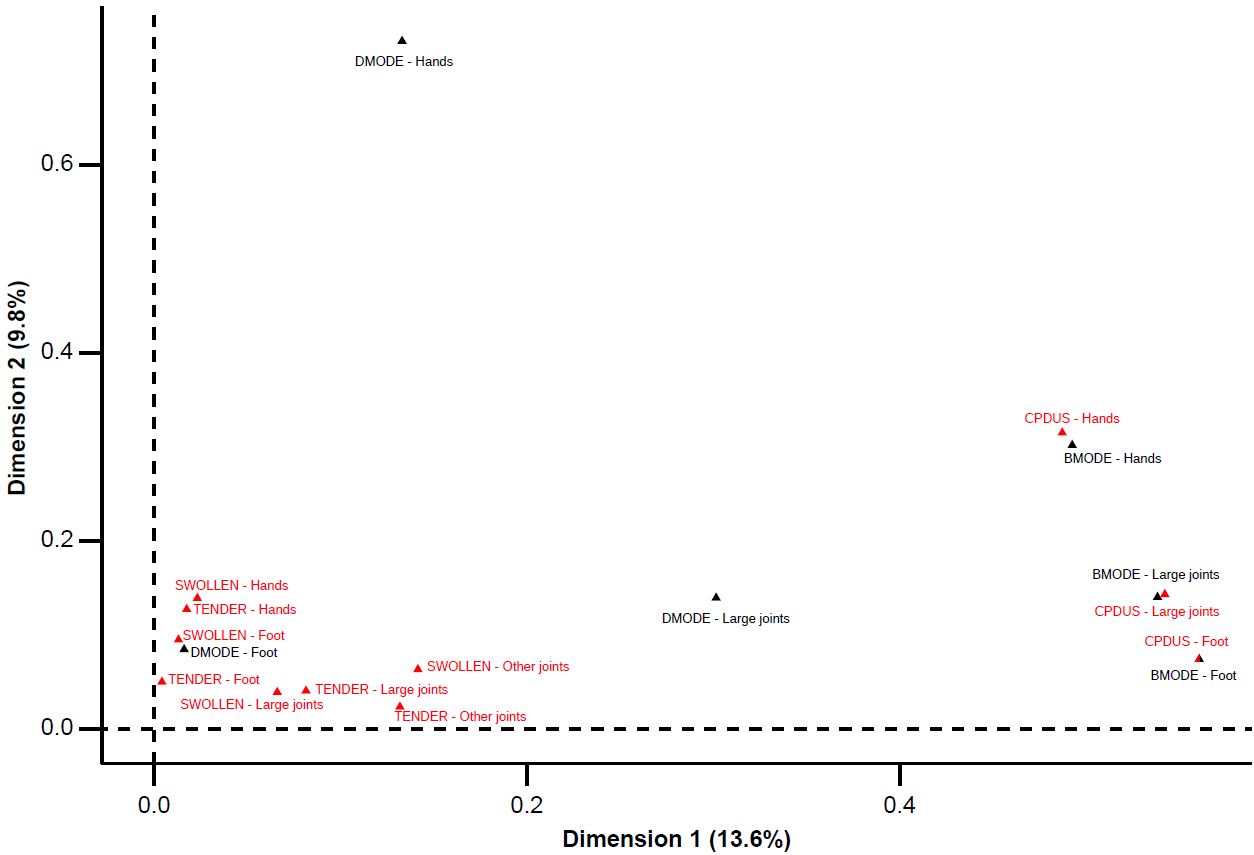


**E) Variable projection according to MFA**


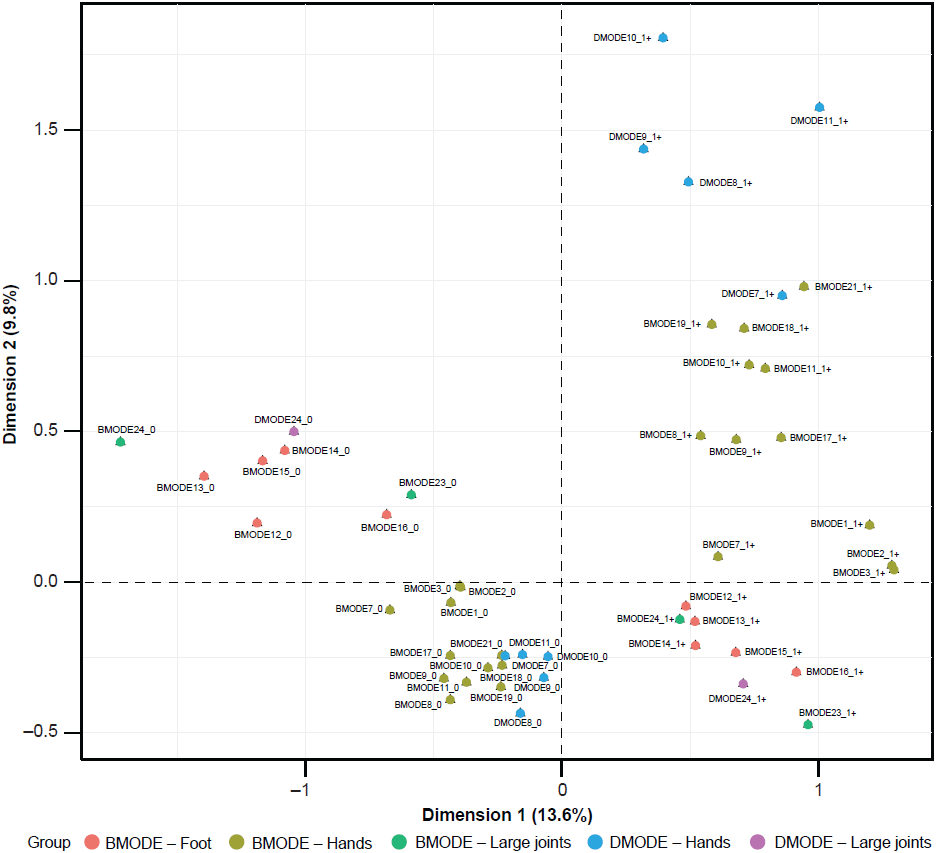


## **Supplementary Fig. S3** Analyses contributing to defining cluster numbers. A) Inter-cluster inertia gains applied in MFA. The number on the x-axis denotes the transition from 1 to 2 clusters, etc. B) Clustered patient factor map. Each patient is positioned according to the first two main dimensions of MFA as a point with color and shape following the cluster phenotype. The 95% ellipses around the barycenter (mean) MFA coordinates of cluster are calculated and displayed. Patients in clusters 2 and 3 are positioned on the right of the graph (along the horizontal axis), and on the left for cluster 1, which depicts the influence of B-mode in foot, large joints, and hands. Cluster 2 patients are at the bottom of the graph (along the vertical axis) compared to cluster 3 on top for the influence of PD-mode and B-mode in the hand.

MFA, multiple factor analysis; PD, power Doppler

**A) Inter-cluster inertia gains applied in MFA**


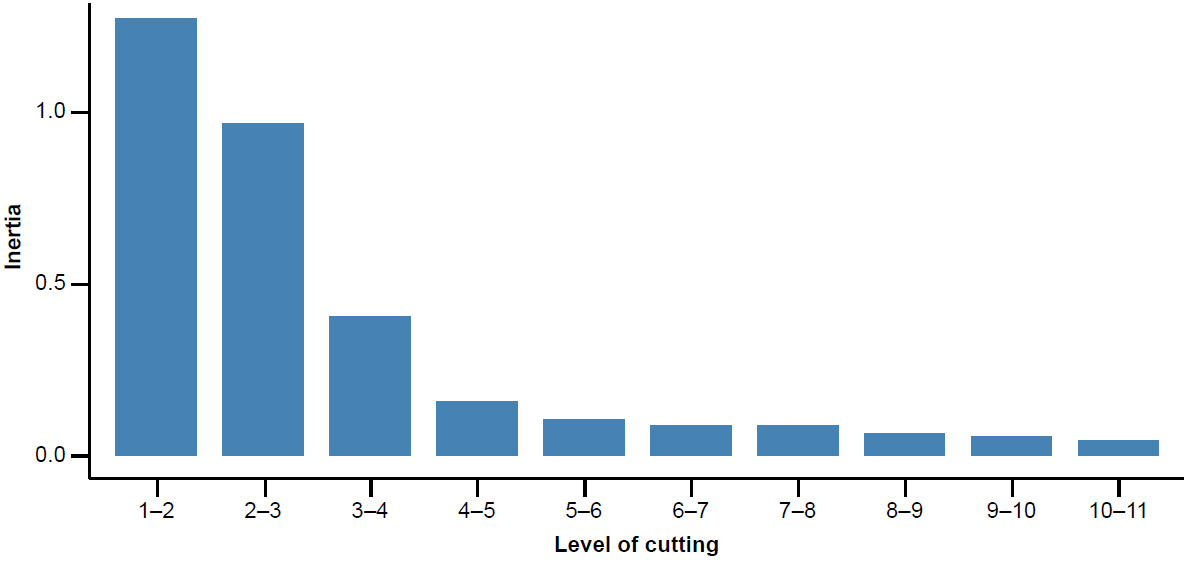


**B) Clustered patient factor map**


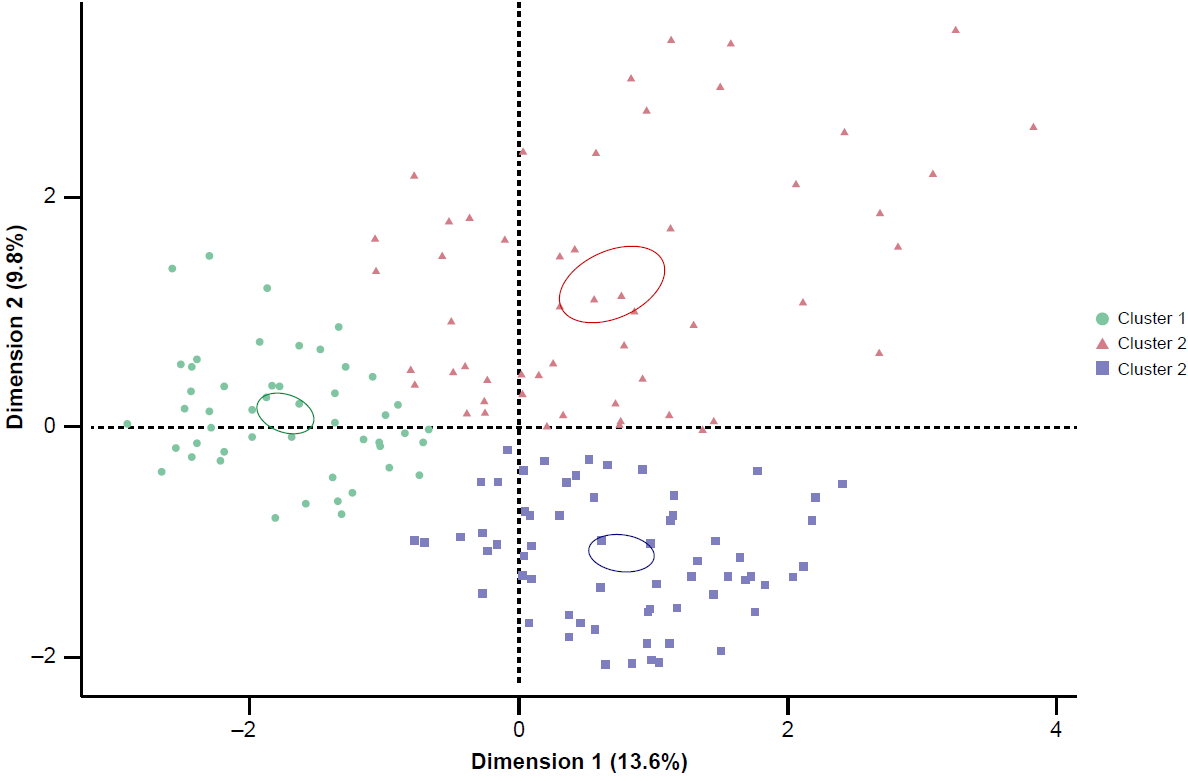


.

## Supplementary Fig. S4. Proportion of enthesitis by grade of severity, treatment arm and cluster (identified by post-hoc analysis on baseline B-mode and PD signal core components of the composite PDUS at joint level) at baseline and week 12. A) Cluster 1. B) Cluster 2. C) Cluster 3.

PD, power Doppler; PDUS, power Doppler ultrasound.

**A) Cluster 1**


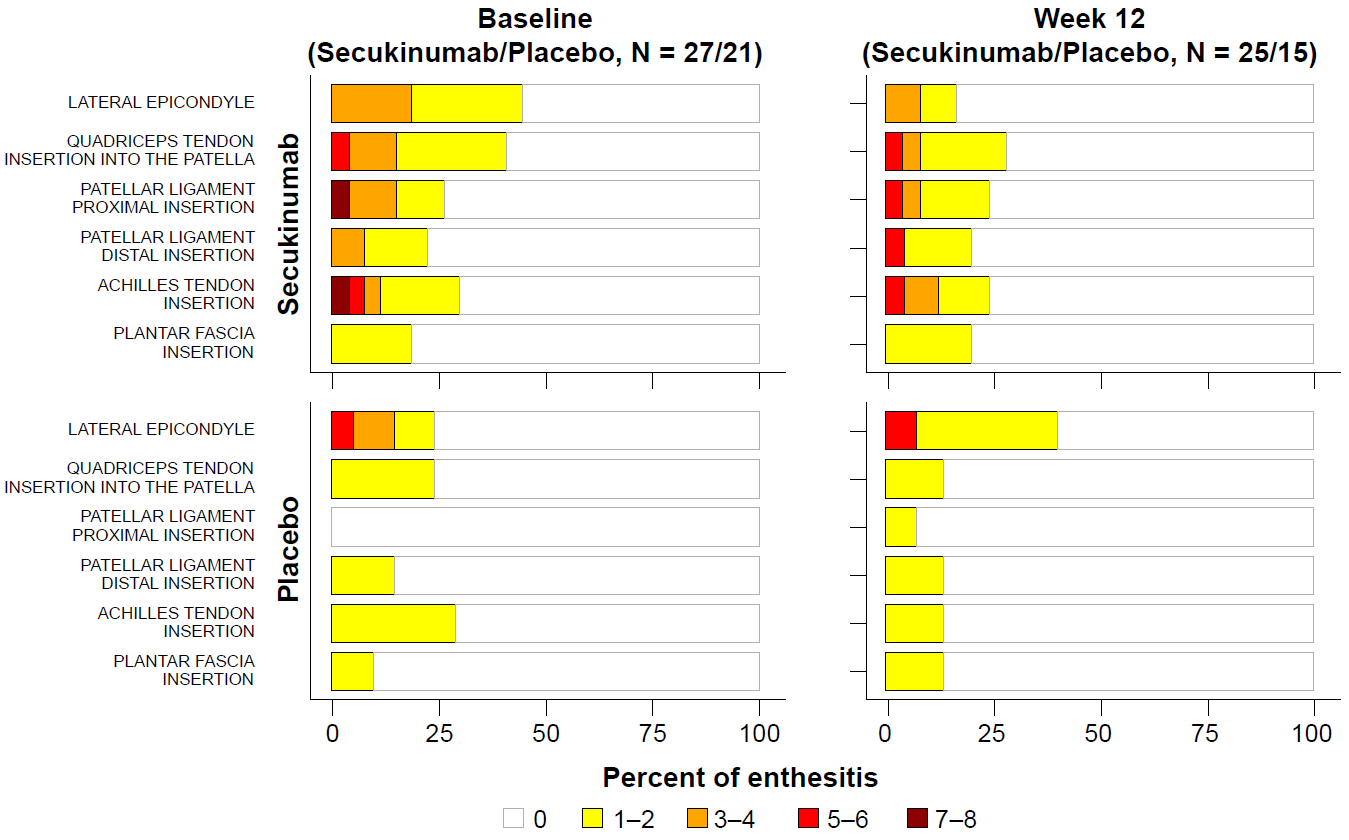


**B) Cluster 2**


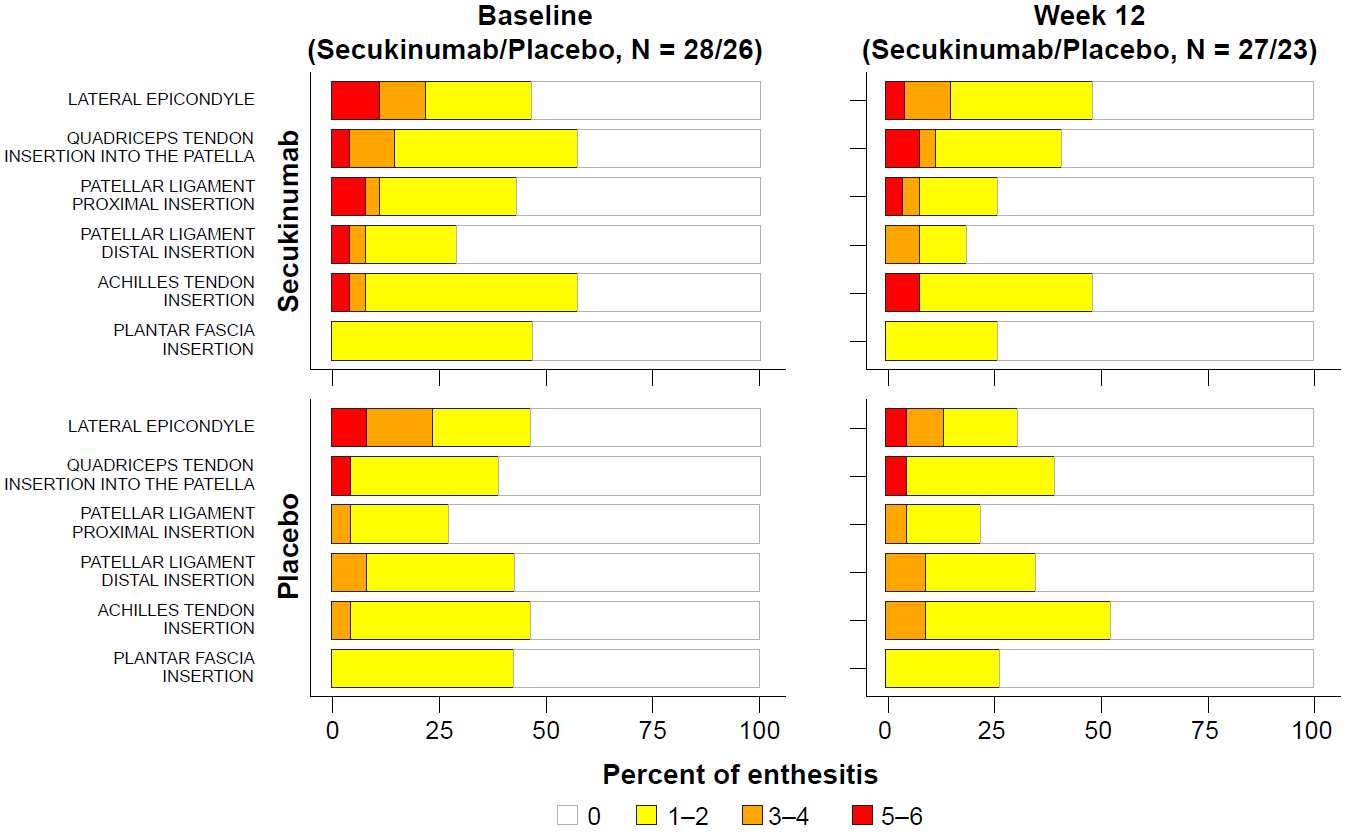


**C) Cluster 3**


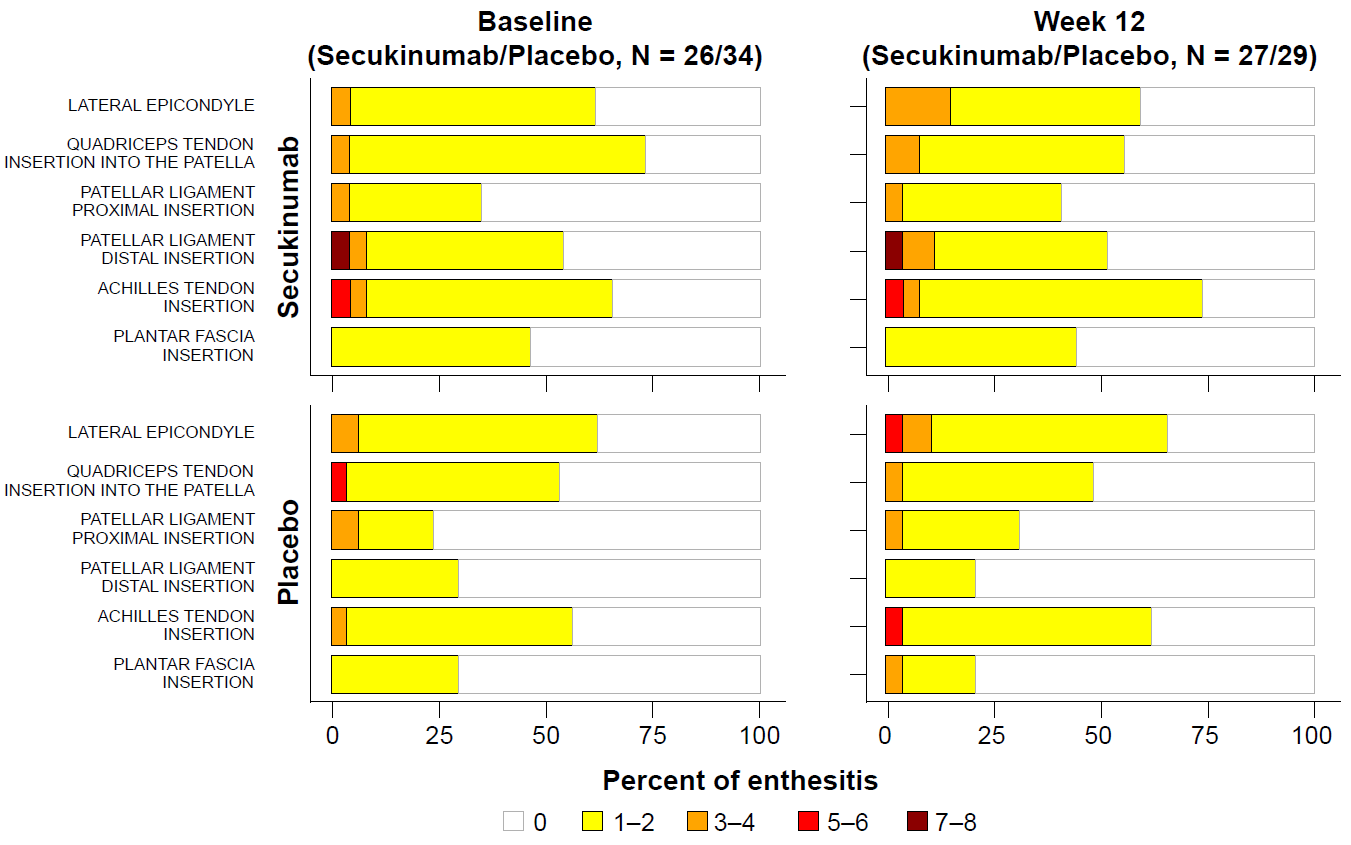


## **Supplementary Fig. S5.** Change from baseline to week 12 in PDUS-detected synovitis by treatment arm and cluster (identified by post-hoc analysis on baseline B-mode and PD-mode core components of the composite PDUS at joint level). A) Cluster 1. B) Cluster 3. DIP, distal interphalangeal joint; MCP, metacarpophalangeal; MTP, metatarsophalangeal; PD, power Doppler; PDUS, power Doppler ultrasound; PIP, proximal interphalangeal.

**A) Cluster 1**


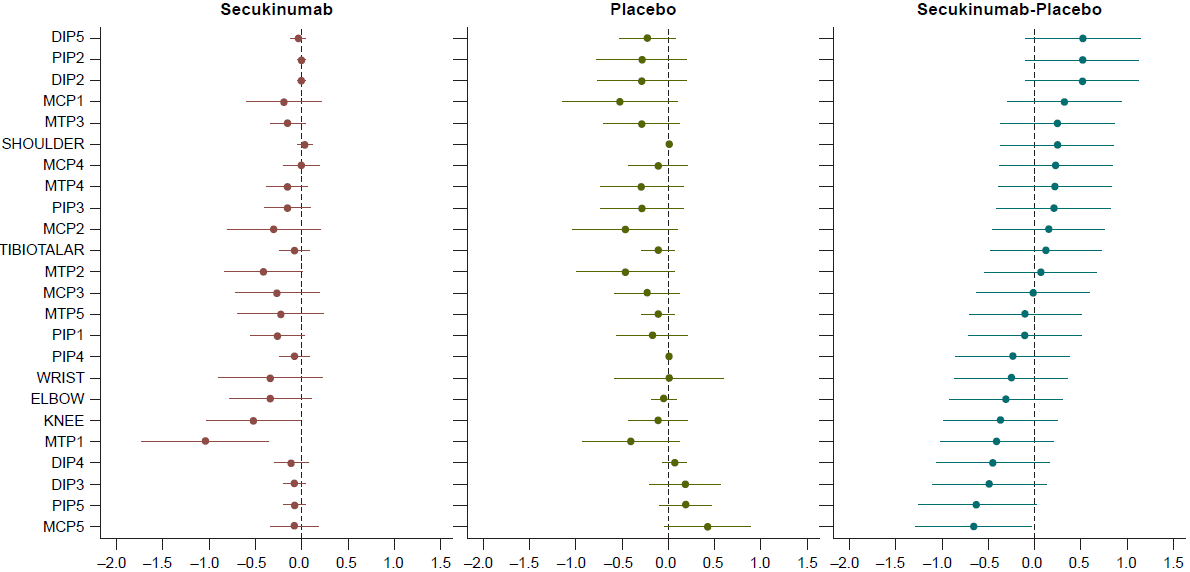


**B) Cluster 3**


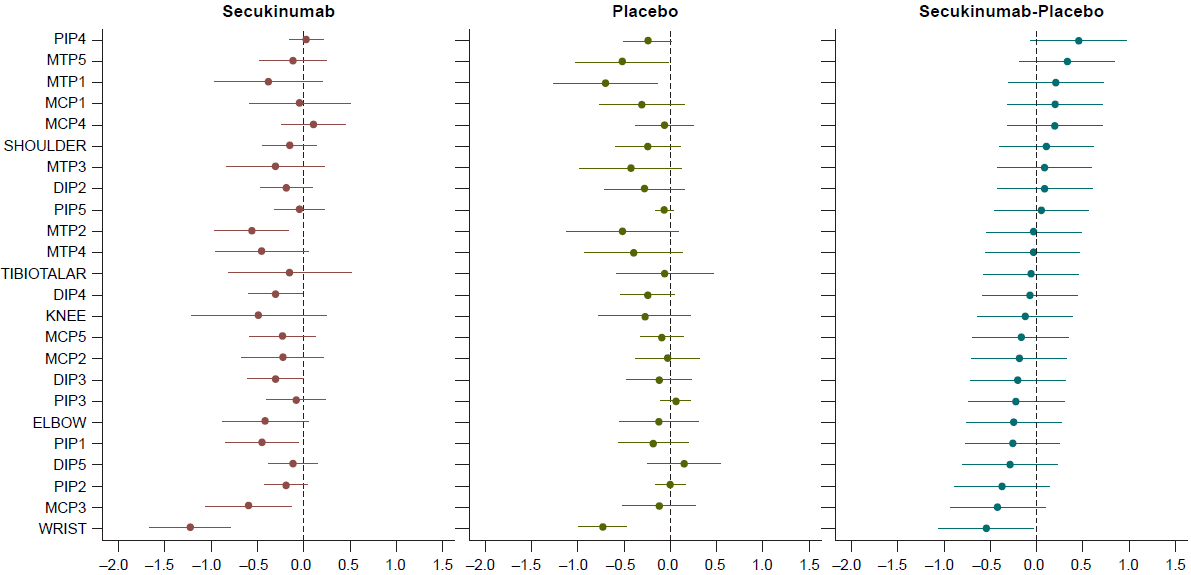


# Supplementary Tables

## Supplementary Table S1 Ultrasound scoring system for B-mode and PD signal at joint level

| B-mode: inflammatory or active synovial hypertrophy | |
| --- | --- |
| Grade 0 | No hypoechoic synovial thickening |
| Grade 1 | Minimal hypoechoic synovial thickening filling the angle between the periarticular bones, without bulging over the line linking tops of the bones |
| Grade 2 | Hypoechoic synovial thickening bulging over the line linking tops of the periarticular bones but without extension along the bone diaphysis |
| Grade 3 | Hypoechoic synovial thickening bulging over the line linking tops of the periarticular bones and with extension to at least one of the bone diaphysis |
| PD signal | |
| Grade 0 | No flow (PD signal) in the synovium |
| Grade 1 | Up to three single spots signals or up to two confluent spots or one confluent spot plus up to two single spots |
| Grade 2 | Vessel signals in less than half of the area of the synovium (<50%) |
| Grade 3 | Vessel signals in more than half of the area of the synovium (>50%) |

Grades: 0, normal joint; 1, minimal synovitis; 2, moderate synovitis; 3, severe synovitis. PD: power Doppler; PDUS: power Doppler ultrasonography.

## Supplementary Table S2 Severity grade of enthesitis

| **Severity scoring** | **Morphological (B mode)** | **Inflammation (Doppler)** |
| --- | --- | --- |
| **0** | No abnormalities | 0 Doppler signal |
| **1** | Hypoechogenicity alone | 1 or 2 Doppler spots at cortical insertion |
| **2** | Thickening and hypoechogenicity plus calcifications/enthesophytes | More than 2 Doppler spots at cortical insertion and up to 2 mm from cortical bone |
| **3** | Thickening and hypoechogenicity plus calcifications/enthesophytes AND erosions | Extensive Doppler signal at cortical insertion |
| **Definitions of various elements of enthesitis:** | | |
| **Hypoechogenicity:** A lack of the homogeneous fibrillar pattern with loss of the tightly packed echogenic lines after correcting for anisotropy. | | |
| **Thickening (increased thickening of the enthesis):** Increased thickness of the tendon/ligament/capsule insertion into the bone, as compared with the body of the tendon/ligament/capsule, with or without blurring of the tendon/ligament/capsule margins. | | |
| **Enthesophytes:** A step up of bony prominence at the end of the normal bone contour, seen in two perpendicular planes, with or without acoustic shadow. | | |
| **Calcifications:** Hyperechoic (bright) foci consistent with calcific deposits, with or without acoustic shadow, seen in two perpendicular planes, detected at the tendon insertion into the bone (i.e., enthesis). | | |
| **Erosion:** A cortical breakage with a step-down contour defect, seen in two perpendicular planes, at the insertion of the enthesis to the bone, according to the OMERACT definition. | | |
| **The Doppler signal:** The Doppler signal at the enthesis was defined as Doppler activity approximately 2 mm near the bony cortex. The Doppler signal must be at the enthesis, different from reflecting surface artifact or nutrition vessel signal, with or without cortical irregularities, erosions, or enthesophytes. | | |

## Supplementary Table S3 OMERACT Definition for PDUS enthesitis: at enthesis level and at patient level

| **Score** | **Definition (activity and structure)** |
| --- | --- |
| **OMERACT enthesitis score  (at enthesis level)**  **Score range** | PD signal (range 0–3) + Gray Scale (B-mode, range 0–1)  0–4 |
| **Global OMERACT enthesitis score (at patient level)**  **Score range** | Sum over 6 sites scored bilaterally  0–48 |

At each visit, the inflammatory and structural components of all affected enthesis sites were scored. The sum of site scores comprises the global enthesitis score at patient level.

OMERACT, Outcome Measures in Rheumatoid Arthritis Clinical Trials; PD, power Doppler; PDUS, power Doppler ultrasonography.

| Supplementary Table S4 Summary of input variables and corresponding block used in the MFA | | | |
| --- | --- | --- | --- |
| **Variable** | **Label** | **Block** | **Group** |
| BMODE12 | B MODE MTP1 | BMODE - Foot | Active |
| BMODE13 | B MODE MTP2 | BMODE - Foot | Active |
| BMODE14 | B MODE MTP3 | BMODE - Foot | Active |
| BMODE15 | B MODE MTP4 | BMODE - Foot | Active |
| BMODE16 | B MODE MTP5 | BMODE - Foot | Active |
| BMODE7 | B MODE MCP1 | BMODE - Hands | Active |
| BMODE8 | B MODE MCP2 | BMODE - Hands | Active |
| BMODE9 | B MODE MCP3 | BMODE - Hands | Active |
| BMODE10 | B MODE MCP4 | BMODE - Hands | Active |
| BMODE11 | B MODE MCP5 | BMODE - Hands | Active |
| BMODE17 | B MODE PIP1 | BMODE - Hands | Active |
| BMODE18 | B MODE PIP2 | BMODE - Hands | Active |
| BMODE19 | B MODE PIP3 | BMODE - Hands | Active |
| BMODE20 | B MODE PIP4 | BMODE - Hands | Active |
| BMODE21 | B MODE PIP5 | BMODE - Hands | Active |
| BMODE1 | B MODE DIP2 | BMODE - Hands | Active |
| BMODE2 | B MODE DIP3 | BMODE - Hands | Active |
| BMODE3 | B MODE DIP4 | BMODE - Hands | Active |
| BMODE4 | B MODE DIP5 | BMODE - Hands | Active |
| BMODE22 | B MODE SHOULDER | BMODE - Large joints | Active |
| BMODE23 | B MODE TIBIOTALAR OR TALONAVICULAR OR SUBTALAR | BMODE - Large joints | Active |
| BMODE24 | B MODE WRIST | BMODE - Large joints | Active |
| BMODE5 | B MODE ELBOW | BMODE - Large joints | Active |
| BMODE6 | B MODE KNEE | BMODE - Large joints | Active |
| DMODE12 | DOPPLER MODE MTP1 | DMODE - Foot | Active |
| DMODE13 | DOPPLER MODE MTP2 | DMODE - Foot | Active |
| DMODE14 | DOPPLER MODE MTP3 | DMODE - Foot | Active |
| DMODE15 | DOPPLER MODE MTP4 | DMODE - Foot | Active |
| DMODE16 | DOPPLER MODE MTP5 | DMODE - Foot | Active |
| DMODE7 | DOPPLER MODE MCP1 | DMODE - Hands | Active |
| DMODE8 | DOPPLER MODE MCP2 | DMODE - Hands | Active |
| DMODE9 | DOPPLER MODE MCP3 | DMODE - Hands | Active |
| DMODE10 | DOPPLER MODE MCP4 | DMODE - Hands | Active |
| DMODE11 | DOPPLER MODE MCP5 | DMODE - Hands | Active |
| DMODE17 | DOPPLER MODE PIP1 | DMODE - Hands | Active |
| DMODE18 | DOPPLER MODE PIP2 | DMODE - Hands | - |
| DMODE19 | DOPPLER MODE PIP3 | DMODE - Hands | - |
| DMODE20 | DOPPLER MODE PIP4 | DMODE - Hands | - |
| DMODE21 | DOPPLER MODE PIP5 | DMODE - Hands | - |
| DMODE1 | DOPPLER MODE DIP2 | DMODE - Hands | - |
| DMODE2 | DOPPLER MODE DIP3 | DMODE - Hands | - |
| DMODE3 | DOPPLER MODE DIP4 | DMODE - Hands | - |
| DMODE4 | DOPPLER MODE DIP5 | DMODE - Hands | - |
| DMODE22 | DOPPLER MODE SHOULDER | DMODE - Large joints | - |
| DMODE23 | DOPPLER MODE TIBIOTALAR OR TALONAVICULAR OR SUBTALAR | DMODE - Large joints | - |
| DMODE24 | DOPPLER MODE WRIST | DMODE - Large joints | Active |
| DMODE5 | DOPPLER MODE ELBOW | DMODE - Large joints | Active |
| DMODE6 | DOPPLER MODE KNEE | DMODE - Large joints | Active |
| SWOJNT19 | SWOLLEN JOINT FOR PSA MTP1 | SWOLLEN - Foot | Supplementary |
| SWOJNT20 | SWOLLEN JOINT FOR PSA MTP2 | SWOLLEN - Foot | Supplementary |
| SWOJNT21 | SWOLLEN JOINT FOR PSA MTP3 | SWOLLEN - Foot | Supplementary |
| SWOJNT22 | SWOLLEN JOINT FOR PSA MTP4 | SWOLLEN - Foot | Supplementary |
| SWOJNT23 | SWOLLEN JOINT FOR PSA MTP5 | SWOLLEN - Foot | Supplementary |
| SWOJNT25 | SWOLLEN JOINT FOR PSA PIP1 (TOE) | SWOLLEN - Foot | Supplementary |
| SWOJNT27 | SWOLLEN JOINT FOR PSA PIP2 (TOE) | SWOLLEN - Foot | Supplementary |
| SWOJNT29 | SWOLLEN JOINT FOR PSA PIP3 (TOE) | SWOLLEN - Foot | Supplementary |
| SWOJNT31 | SWOLLEN JOINT FOR PSA PIP4 (TOE) | SWOLLEN - Foot | Supplementary |
| SWOJNT33 | SWOLLEN JOINT FOR PSA PIP5 (TOE) | SWOLLEN - Foot | Supplementary |
| SWOJNT5 | SWOLLEN JOINT FOR PSA DIP2 (FOOT) | SWOLLEN - Foot | Supplementary |
| SWOJNT7 | SWOLLEN JOINT FOR PSA DIP3 (FOOT) | SWOLLEN - Foot | - |
| SWOJNT9 | SWOLLEN JOINT FOR PSA DIP4 (FOOT) | SWOLLEN - Foot | Supplementary |
| SWOJNT11 | SWOLLEN JOINT FOR PSA DIP5 (FOOT) | SWOLLEN - Foot | - |
| SWOJNT14 | SWOLLEN JOINT FOR PSA MCP1 | SWOLLEN - Hands | Supplementary |
| SWOJNT15 | SWOLLEN JOINT FOR PSA MCP2 | SWOLLEN - Hands | Supplementary |
| SWOJNT16 | SWOLLEN JOINT FOR PSA MCP3 | SWOLLEN - Hands | Supplementary |
| SWOJNT17 | SWOLLEN JOINT FOR PSA MCP4 | SWOLLEN - Hands | Supplementary |
| SWOJNT18 | SWOLLEN JOINT FOR PSA MCP5 | SWOLLEN - Hands | Supplementary |
| SWOJNT24 | SWOLLEN JOINT FOR PSA PIP1 | SWOLLEN - Hands | Supplementary |
| SWOJNT26 | SWOLLEN JOINT FOR PSA PIP2 | SWOLLEN - Hands | Supplementary |
| SWOJNT28 | SWOLLEN JOINT FOR PSA PIP3 | SWOLLEN - Hands | Supplementary |
| SWOJNT30 | SWOLLEN JOINT FOR PSA PIP4 | SWOLLEN - Hands | Supplementary |
| SWOJNT32 | SWOLLEN JOINT FOR PSA PIP5 | SWOLLEN - Hands | Supplementary |
| SWOJNT4 | SWOLLEN JOINT FOR PSA DIP2 | SWOLLEN - Hands | Supplementary |
| SWOJNT6 | SWOLLEN JOINT FOR PSA DIP3 | SWOLLEN - Hands | Supplementary |
| SWOJNT8 | SWOLLEN JOINT FOR PSA DIP4 | SWOLLEN - Hands | Supplementary |
| SWOJNT10 | SWOLLEN JOINT FOR PSA DIP5 | SWOLLEN - Hands | Supplementary |
| SWOJNT1 | SWOLLEN JOINT FOR PSA ANKLE JOINT | SWOLLEN - Large joints | Supplementary |
| SWOJNT12 | SWOLLEN JOINT FOR PSA ELBOW JOINT | SWOLLEN - Large joints | Supplementary |
| SWOJNT13 | SWOLLEN JOINT FOR PSA KNEE JOINT | SWOLLEN - Large joints | Supplementary |
| SWOJNT34 | SWOLLEN JOINT FOR PSA SHOULDER | SWOLLEN - Large joints | Supplementary |
| SWOJNT38 | SWOLLEN JOINT FOR PSA WRIST JOINT | SWOLLEN - Large joints | Supplementary |
| SWOJNT2 | SWOLLEN JOINT FOR PSA ACROMIOCLAVICULAR JOINT | SWOLLEN - Other joints | - |
| SWOJNT3 | SWOLLEN JOINT FOR PSA CMC1 | SWOLLEN - Other joints | Supplementary |
| SWOJNT35 | SWOLLEN JOINT FOR PSA STERNOCLAVICULAR JOINT | SWOLLEN - Other joints | Supplementary |
| SWOJNT36 | SWOLLEN JOINT FOR PSA TARSUS | SWOLLEN - Other joints | Supplementary |
| SWOJNT37 | SWOLLEN JOINT FOR PSA TEMPOROMANDIBULAR JOINT | SWOLLEN - Other joints | Supplementary |
| TENJNT20 | TENDER JOINT FOR PSA MTP1 | TENDER - Foot | Supplementary |
| TENJNT21 | TENDER JOINT FOR PSA MTP2 | TENDER - Foot | Supplementary |
| TENJNT22 | TENDER JOINT FOR PSA MTP3 | TENDER - Foot | Supplementary |
| TENJNT23 | TENDER JOINT FOR PSA MTP4 | TENDER - Foot | Supplementary |
| TENJNT24 | TENDER JOINT FOR PSA MTP5 | TENDER - Foot | Supplementary |
| TENJNT26 | TENDER JOINT FOR PSA PIP1 (TOE) | TENDER - Foot | Supplementary |
| TENJNT28 | TENDER JOINT FOR PSA PIP2 (TOE) | TENDER - Foot | Supplementary |
| TENJNT30 | TENDER JOINT FOR PSA PIP3 (TOE) | TENDER - Foot | Supplementary |
| TENJNT32 | TENDER JOINT FOR PSA PIP4 (TOE) | TENDER - Foot | Supplementary |
| TENJNT34 | TENDER JOINT FOR PSA PIP5 (TOE) | TENDER - Foot | Supplementary |
| TENJNT5 | TENDER JOINT FOR PSA DIP2 (FOOT) | TENDER - Foot | Supplementary |
| TENJNT7 | TENDER JOINT FOR PSA DIP3 (FOOT) | TENDER - Foot | Supplementary |
| TENJNT9 | TENDER JOINT FOR PSA DIP4 (FOOT) | TENDER - Foot | Supplementary |
| TENJNT11 | TENDER JOINT FOR PSA DIP5 (FOOT) | TENDER - Foot | - |
| TENJNT15 | TENDER JOINT FOR PSA MCP1 | TENDER - Hands | Supplementary |
| TENJNT16 | TENDER JOINT FOR PSA MCP2 | TENDER - Hands | Supplementary |
| TENJNT17 | TENDER JOINT FOR PSA MCP3 | TENDER - Hands | Supplementary |
| TENJNT18 | TENDER JOINT FOR PSA MCP4 | TENDER - Hands | Supplementary |
| TENJNT19 | TENDER JOINT FOR PSA MCP5 | TENDER - Hands | Supplementary |
| TENJNT25 | TENDER JOINT FOR PSA PIP1 | TENDER - Hands | Supplementary |
| TENJNT27 | TENDER JOINT FOR PSA PIP2 | TENDER - Hands | Supplementary |
| TENJNT29 | TENDER JOINT FOR PSA PIP3 | TENDER - Hands | Supplementary |
| TENJNT31 | TENDER JOINT FOR PSA PIP4 | TENDER - Hands | Supplementary |
| TENJNT33 | TENDER JOINT FOR PSA PIP5 | TENDER - Hands | Supplementary |
| TENJNT4 | TENDER JOINT FOR PSA DIP2 | TENDER - Hands | Supplementary |
| TENJNT6 | TENDER JOINT FOR PSA DIP3 | TENDER - Hands | Supplementary |
| TENJNT8 | TENDER JOINT FOR PSA DIP4 | TENDER - Hands | Supplementary |
| TENJNT10 | TENDER JOINT FOR PSA DIP5 | TENDER - Hands | Supplementary |
| TENJNT1 | TENDER JOINT FOR PSA ANKLE JOINT | TENDER - Large joints | Supplementary |
| TENJNT12 | TENDER JOINT FOR PSA ELBOW JOINT | TENDER - Large joints | Supplementary |
| TENJNT13 | TENDER JOINT FOR PSA HIP | TENDER - Large joints | Supplementary |
| TENJNT14 | TENDER JOINT FOR PSA KNEE JOINT | TENDER - Large joints | Supplementary |
| TENJNT35 | TENDER JOINT FOR PSA SHOULDER | TENDER - Large joints | Supplementary |
| TENJNT39 | TENDER JOINT FOR PSA WRIST JOINT | TENDER - Large joints | Supplementary |
| TENJNT2 | TENDER JOINT FOR PSA ACROMIOCLAVICULAR JOINT | TENDER - Other joints | Supplementary |
| TENJNT3 | TENDER JOINT FOR PSA CMC1 | TENDER - Other joints | Supplementary |
| TENJNT36 | TENDER JOINT FOR PSA STERNOCLAVICULAR JOINT | TENDER - Other joints | Supplementary |
| TENJNT37 | TENDER JOINT FOR PSA TARSUS | TENDER - Other joints | Supplementary |
| TENJNT38 | TENDER JOINT FOR PSA TEMPOROMANDIBULAR JOINT | TENDER - Other joints | Supplementary |
| CPDUS12 | COMPOSITE PDUS MTP1 | CPDUS - Foot | Supplementary |
| CPDUS13 | COMPOSITE PDUS MTP2 | CPDUS - Foot | Supplementary |
| CPDUS14 | COMPOSITE PDUS MTP3 | CPDUS - Foot | Supplementary |
| CPDUS15 | COMPOSITE PDUS MTP4 | CPDUS - Foot | Supplementary |
| CPDUS16 | COMPOSITE PDUS MTP5 | CPDUS - Foot | Supplementary |
| CPDUS7 | COMPOSITE PDUS MCP1 | CPDUS - Hands | Supplementary |
| CPDUS8 | COMPOSITE PDUS MCP2 | CPDUS - Hands | Supplementary |
| CPDUS9 | COMPOSITE PDUS MCP3 | CPDUS - Hands | Supplementary |
| CPDUS10 | COMPOSITE PDUS MCP4 | CPDUS - Hands | Supplementary |
| CPDUS11 | COMPOSITE PDUS MCP5 | CPDUS - Hands | Supplementary |
| CPDUS17 | COMPOSITE PDUS PIP1 | CPDUS - Hands | Supplementary |
| CPDUS18 | COMPOSITE PDUS PIP2 | CPDUS - Hands | Supplementary |
| CPDUS19 | COMPOSITE PDUS PIP3 | CPDUS - Hands | Supplementary |
| CPDUS20 | COMPOSITE PDUS PIP4 | CPDUS - Hands | Supplementary |
| CPDUS21 | COMPOSITE PDUS PIP5 | CPDUS - Hands | Supplementary |
| CPDUS1 | COMPOSITE PDUS DIP2 | CPDUS - Hands | Supplementary |
| CPDUS2 | COMPOSITE PDUS DIP3 | CPDUS - Hands | Supplementary |
| CPDUS3 | COMPOSITE PDUS DIP4 | CPDUS - Hands | Supplementary |
| CPDUS4 | COMPOSITE PDUS DIP5 | CPDUS - Hands | Supplementary |
| CPDUS22 | COMPOSITE PDUS SHOULDER | CPDUS - Large joints | Supplementary |
| CPDUS23 | COMPOSITE PDUS TIBIOTALAR OR TALONAVICULAR OR SUBTALAR | CPDUS - Large joints | Supplementary |
| CPDUS24 | COMPOSITE PDUS WRIST | CPDUS - Large joints | Supplementary |
| CPDUS5 | COMPOSITE PDUS ELBOW | CPDUS - Large joints | Supplementary |
| CPDUS6 | COMPOSITE PDUS KNEE | CPDUS - Large joints | Supplementary |
|  | | | |

-, variables filtered out for the MFA analysis; BMODE, GLOESS synovial hypertrophy (B-mode); DIP, distal interphalangeal; DMODE, GLOESS PD signal; CPDUS, GLOESS PDUS score; MCP, metacarpophalangeal; MFA, multiple factor analysis; MTP, metatarsophalangeal; PD, power Doppler; PDUS, power Doppler ultrasound; PIP, proximal interphalangeal.

## Supplementary Table S5 Proportion of joint synovitis based on composite PDUS score in different clusters

|  | Cluster 1  (n = 49) | Cluster 2 (n = 54) | Cluster 3  (n = 63) | Total  (N = 166) |
| --- | --- | --- | --- | --- |
| **Large joints** | | | | |
| Shoulder | 1 (2%) | 13 (24%) | 9 (14%) | 23 (14%) |
| Tibiotalar or talonavicular or subtalar | 5 (10%) | 18 (33%) | 40 (63%) | 63 (38%) |
| Wrist | 21 (43%) | 48 (89%) | 63 (100%) | 132 (80%) |
| Elbow | 7 (14%) | 20 (37%) | 40 (63%) | 67 (40%) |
| Knee | 14 (29%) | 29 (54%) | 54 (86%) | 97 (58%) |
| **Hands and feet joints** |  |  |  |  |
| MCP | 30 (61%) | 52 (96%) | 48 (76%) | 130 (78%) |
| PIP | 21 (43%) | 41 (76%) | 32 (51%) | 94 (57%) |
| DIP | 9 (18%) | 28 (52%) | 38 (60%) | 75 (45%) |
| MTP | 31 (63%) | 52 (96%) | 62 (98%) | 145 (87%) |

DIP, distal interphalangeal; MCP, metacarpophalangeal; MTP, metatarsophalangeal; PDUS, power Doppler ultrasound; PIP, proximal interphalangeal.

## Supplementary Table S6 Mean (SE) change in PD signal and synovial hypertrophy (B-mode) from baseline to week 12 by ultrasound cluster and treatment

| **Treatment** | **Cluster 1 (n = 49)** | **Cluster 2 (n = 54)** | **Cluster 3  (n = 63)** |
| --- | --- | --- | --- |
| **PD signal** |  |  |  |
| Secukinumab | −3 (0.8) | −6. (0.8) | −3 (0.9) |
| Placebo | −3 (1.0) | −2. (0.8) | −2 (0.8) |
| Difference (secukinumab – placebo) | 0 (1.2) | −4 (1.1) | −1 (0.9) |
| **Synovial hypertrophy (B-mode)** |  |  |  |
| Secukinumab | −9 (1.6) | −11 (1.4) | −7 (1.6) |
| Placebo | −7 (1.9) | −5 (1.5) | −6 (1.4) |
| Difference (secukinumab – placebo) | −2 (2.1) | −6 (2.0) | −1 (1.8) |

PD, power Doppler; SE, standard error.
